# Alpha-N-acetylglucosaminidase (NAGLU) inactivation causes morphological damage to the ultrastructural appearance of epididymal mononuclear phagocytes (eMPs) and epithelial cells of the epididymis in adult mice

**DOI:** 10.1371/journal.pone.0359319

**Published:** 2026-09-25

**Authors:** Lorena Carvelli, Louis Hermo, Daniel G. Cyr, Mary Gregory, Alexey V. Pshezhetsky, Carlos R. Morales

**Affiliations:** 1 Department of Anatomy and Cell Biology, McGill University, Montreal, Quebec, Canada; 2 IHEM-CONICET, Universidad Nacional de Cuyo, Mendoza, Argentina; 3 Facultad de Ciencias Exactas y Naturales, Universidad Nacional de Cuyo, Mendoza, Argentina; 4 CHU Sainte-Justine Research Centre, Department of Pediatrics, University of Montreal, Montreal, Quebec, Canada; 5 INRS-Institut Armand Frappier, Université du Québec, Laval, Quebec, Canada; University of Hyderabad, INDIA

## Abstract

Heparan sulfate (HS) a glycosaminoglycan family of carbohydrates, is covalently attached to core proteins forming heparan sulfate proteoglycans (HSPGs) which are found on the surface of cells. Following endocytosis, HSPGs are degraded within lysosomes by several enzymes, including α-N-acetylglucosaminidase (NAGLU). Absence of NAGLU leads to Mucopolysaccharidosis III type B (Sanfilippo type B), a lysosomal storage disease, which in *Naglu-/-* mice was suggested to result in infertility. Since endocytosis is a feature of epididymal epithelial and immune cells, a plausible explanation could be excessive accumulation of lysosomes in these cells in 7-month-old mice. EM analysis revealed a massive accumulation of pale lysosomes (LM-IHC/LAMP2 + /cathepsin B+) of varying shapes and sizes in the cytoplasm of principal cells, unlike the small dense spherical lysosomes of wild type mice. Their presence had a profound effect on the integrity of organelles such as the ER, Golgi, mitochondria and nucleus. In addition, the epithelium demonstrated sporadic large, highly vacuolated cells, identified as CD68+ and MOMA+ epididymal mononuclear phagocytes (eMPs), with a cytoplasm dominated by pale lysosomes, some of gigantic size. The plethora of lysosomes overwhelming the cytoplasm suggested an ongoing phagocytic activity to protect the epithelium despite the excessive disruption of their organelles. A distinguishing feature of the content of lysosomes of eMPs was the presence of aggregates of small flattened membranous discs aligned parallel to one another, referred to as zebra bodies. The latter had the appearance of mitochondrial cristae of principal cells and were reactive for a mitochondrial marker, suggesting a phagocytic activity for eMPs of degenerating principal cells. Taken together, our data demonstrates that the accumulation of lysosomes of epithelial cells and eMPs dramatically altered their structural appearance in the absence of NAGLU inducing an inactivation of their critical role in supporting a proper epithelial environment for sperm protection and maturation.

## Introduction

The mammalian male reproductive tract is formed in part by the testis, efferent ducts and epididymis which in their own domains are critical for successful reproduction. The seminiferous epithelium of the adult testis consists of generations of germ cells that divide, undergo meiosis and metamorphosis, and gradually develop into maturing sperm with the support of nondividing Sertoli cells [1–4]. The interstitial space between seminiferous tubules houses the steroid producing Leydig cells as well as a colony of resident and incoming macrophages [5,6]. Sperm produced in the testis exit via a series of small ducts referred to as the efferent ducts which serve as a passageway for entry into the epididymis [7].

The epididymis is a highly tortuous duct that is compacted upon itself and enclosed in a connective tissue capsule. While the testis is involved in the formation and development of sperm, the role of the epididymis is to provide sperm with their capability to fertilize the ovum [8–12]. Throughout its entire length, it is lined by a pseudostratified epithelium composed primarily of columnar principal and clear cells that extend from the base to the lumen, as well as basal cells lodged at the base of the epithelium [8,13–15]. Based on structural features and specific functions of its epithelial cells, the duct is divided into 4 major regions, with each containing distinct subdivisions [16]. These regions are defined as the initial segment, caput, corpus and cauda. In addition to its major role in sperm maturation, the epididymis also serves as a mode of concentration, transport and storage depot for sperm [8,9,13,17,18].

Additionally, the epididymis displays along its length a gradually changing epithelial architecture that supports the distinct functional demands of each region, and this includes a high demand for immune surveillance with a dense population of resident immune cells. Such cells were initially described as wandering or circulating halo cells [19,20] characterized morphologically as monocyte/macrophages (M/M) and lymphocytes [8,13,21–23]. In 2011, a dramatic change in the makeup of the epithelial architecture occurred when Da Silva et al. [24] defined a novel population of epididymal mononuclear phagocytes (eMPs) by confocal microscopy consisting of resident F4/80^+^ (M/M) and CD11c^+^/CX3CR1^+^ dendritic cells. Over the years, multiple subsets of the eMP family have been defined possessing distinct functions related to tissue homeostasis and pathogen-specific immunity [25–30].

Glycoproteins and glycolipids form a dense layer that coats the surface of cells, referred to as the glycocalyx, composed of various polysaccharides covalently linked to lipids or proteins [31,32]Heparan sulfate (HS), belonging to the glycosaminoglycan (GAG) family of carbohydrates, is covalently attached to core proteins forming heparan sulfate proteoglycans (HSPGs). The latter are ubiquitously present in different tissues and organs of the body including the male reproductive tract, indicating a multitude of critical biological roles they play within an organism [33,34]. This includes not only cell surface coats, but an integral basement membrane constituent and scaffolding component of the extracellular matrix [35]. HSPGs interact with a wide array of ligands and cognate receptors and act as crucial components of protein binding both within and between cells [36,37].

Lysosomal storage disorders (LSDs) comprise a heterogeneous group of rare inherited metabolic diseases characterized by accumulation of macromolecules inside lysosomes caused by deficiencies in lysosomal enzymes and leading to altered recycling of macromolecules and impairment of the endolysosomal system [38–40]. Mucopolysaccharidoses are a group of LSDs accounting for approximately 30% of all cases and arise from mutations in genes involved in glycosaminoglycans degradation [41,42]. Under normal conditions, HS degradation following endocytosis proceeds in a stepwise fashion with the help of several enzymes [43]. Based on which enzyme is deficient, four different subtypes of MPS type III (MPS IIIA-D) diseases have been recognized [44].

Mucopolysaccharidosis III B (MPS III B, Sanfilippo syndrome type B) is a lysosomal storage disorder caused by mutation of the *Naglu* gene, α-N-acetylglucosaminidase (EC 3.2.1.50), involved in the degradation of HS [44–46]. NAGLU removes the terminal N-acetyl-D-glucosamine in HS oligosaccharides [47]. Mutations to the *Naglu* gene give rise to MPS IIIB (Sanfilippo syndrome type B) resulting in reduced or absent enzyme activity and preventing complete heparan sulfate degradation and accumulation in lysosomes [48]. In a recent study, we evaluated the role of HSPG degradation in mice deficient in the enzyme heparin-alpha-glucosaminide N-acetyltransferase (HGSNAT), which causes Mucopolysaccharidosis III type C (Sanfilippo type C) [49]. In the present study, we broaden our knowledge of HS degradation by investigating the *Naglu* knockout mouse model. While male infertility has been reported in *Naglu* KO mice from six months of age onward [50], no structural or functional fertility parameters were presented to support this observation. Hence the objective of this study was to examine one component of the male reproductive tract, i.e., the ultrastructural features of the epithelial cells of the testis, efferent ducts and epididymis as potential candidates for the proposed infertility. Given the conservation of testicular and epididymal architecture and immune features across mammals, insights from murine models are relevant for understanding the implications of MPS IIIB disorders in male reproductive tract of human patients.

## Materials and methods

### Animals

Epididymal and testicular tissues of wild-type (C57BL/6) and *Naglu^-/-^* mice were analyzed using light (LM) and transmission electron microscopy (TEM). *Naglu^-/-^* mice (n = 3 KO, 7-month-old) and C57BL/6 wild-type mice (n = 3 WT, 7-month-old) were bred and maintained according to the Canadian Council on Animal Care guidelines. Mice were housed under constant temperature and humidity on a 12-hour light/dark cycle, with food and water provided *ad libitum*. Euthanasia was performed by carbon dioxide inhalation except for animals designated for electron microscopy, as described below. All procedures were approved by the CHU Sainte-Justine Research Ethics Committee (Montreal, QC) and carried out in accordance with the Comité Institutionnel des Bonnes Pratiques Animales en Recherche (CIBPAR, approval number 2022–3453).

### Tissue preparation: Light and electron microscopy

Epididymides and testes were harvested from 7-month-old mice (n = 3 WT, n = 3 KO). Prior to dissection, animals were anesthetized by intraperitoneal injection of sodium pentobarbital (Somnitol, MTC Pharmaceuticals, Hamilton, ON).

### Light microscopy (LM)

The testis, efferent ducts and epididymis of one side of each animal (WT and KO) were removed following clamping and transection of blood vessels adjacent to the rete testis. The testis was cut in cross section, while the efferent ducts were carefully dissected free of surrounding fat and the epididymis sectioned longitudinally to include the initial segment, caput, corpus, and cauda regions. Tissues were placed in Bouin’s fixative (BD Biosciences, Mississauga, ON) for 24 hours, then dehydrated in graded ethanol and embedded in paraffin. Paraffin sections (5 μm) were stained with Periodic Acid-Schiff (PAS) or processed for immunohistochemistry as described below.

### Electron microscopy (EM)

Immediately following removal of these tissues, mice were perfused via the left ventricle with 2.5% glutaraldehyde in 0.1 M sodium cacodylate buffer (pH 7.4) containing 0.05% calcium chloride. Testes, efferent ducts and all four epididymal regions were dissected and cut into ~1 mm^3^ blocks. Samples were post-fixed on ice for several hours and washed overnight at 4 °C in cacodylate buffer. The following day, tissues were post-fixed with 1% osmium tetroxide and 1.5% potassium ferrocyanide, dehydrated in a graded acetone series and embedded in Epon 812 resin (Mecalab Ltd., Montreal, QC). Ultrathin sections (60 nm) were cut with a diamond knife, mounted on copper grids, stained with uranyl acetate (5 min) and lead citrate (3 min), and examined using a FEI Tecnai 12 120 kV Transmission Electron Microscope (TEM). Semithin (5 μm) sections from Epon blocks were stained with toluidine blue and analyzed by light microscopy (Axio Imager 2).

### Light microscopy: Immunohistochemistry, immunofluorescence and TUNEL assay (LM-IHC/IF)

Paraffin sections (5 μm) of testicular and epididymal tissue of WT and KO mice fixed in Bouin’s fixative were deparaffinized in Citrisolv (Decon Laboratories Inc.), rehydrated in graded ethanol, and washed in PBS. Antigen retrieval was performed in 7.1 mM citrate buffer (pH 6.0) using microwave treatment (3 min full power, followed by 7 min at 60% power). After cooling, endogenous peroxidase activity was blocked using a solution of 0.03% hydrogen peroxide and 0.031 M sodium azide (Dako Canada Inc., Mississauga, ON).

Slides were then incubated overnight at 4°C in 50 mM Tris-Cl, pH 7.4 containing 1% Bovine serum albumin (Sigma A7511) with either anti-prosaposin (PSAP, RRID:AB_2792974) [51], anti-cytokeratin 5 (KRT5, Abcam Cat# ab53121, RRID:AB_869889) [52] anti-CD68 (abcam, Cat# ab125212, RRID:AB_10975465) [53] anti-CX3CR1 (BioLegend Cat# 149027, RRID:AB_2565937) [54] or anti-MOMA-2 antibodies (Bio-Rad Cat# MCA519GT, RRID:AB_1102752) [55]

After washing in TBST (0.1% Tween 20 in Tris-buffered saline), sections were incubated with appropriate polymer-HRP-conjugated secondary antibodies (Dako EnVision+ System-HRP, Cat# K4010) for 1 hour at room temperature. Immunoreactivity was revealed using a 2% 3,3-diaminobenzidine (DAB) solution and counterstained with methylene blue. Sections were dehydrated in graded ethanol and Citrisolv and mounted with Permount (Fisher Scientific, Ottawa ON). Negative controls were performed in the absence of the primary antibody. Sections were analyzed by light microscopy (Axio Imager 2).

For indirect immunofluorescence (IF), paraffin sections were processed as described above for deparaffinization, rehydration, washing, and antigen retrieval. Sections were incubated with primary antibodies against SCMAS (anti-ATP synthase C antibody [EPR13907], Abcam, Cat# ab181243), Cathepsin B (anti-Cathepsin B antibody [EPR21033], Abcam, Cat# ab214428), heparan sulfate (anti-Heparan Sulfate, 10E4 epitope, AMSBIO, Cat# 370255), and LAMP2 (Hybridoma Bank, Cat# ABL-93, RRID), followed by incubation with appropriate Alexa dye-conjugated secondary antibodies (Molecular Probes/Invitrogen, Eugene, OR, USA). Sections were mounted using ProLong™ Gold antifade reagent with DAPI (Molecular Probes/Invitrogen, Eugene, OR, USA). Fluorescence images were acquired using a Leica SP8-DLS confocal microscope.

TUNEL labeling was performed on paraffin sections using the DeadEnd™ Fluorometric TUNEL System (Promega, Cat# G3250), according to the manufacturer’s instructions, followed by DAPI counterstaining. Fluorescence images were acquired using a Leica SP8-DLS confocal microscope.

### NAGLU enzymatic activity

NAGLU enzymatic activity was measured in protein homogenates prepared from testis and epididymis of four WT and four *Naglu-/-* mice. NAGLU activity was determined using the fluorogenic substrate 4-Methylumbelliferyl-N-acetyl-α-D-glucosaminide. The fluorescence of released 4-methylumbelliferone was measured using a CLARIOstar microplate reader, and NAGLU activity was calculated from a 4-methylumbelliferone calibration curve. Enzymatic activity was normalized to total protein concentration and expressed as mol/h/mg protein. Data were analyzed using GraphPad Prism version 11.02 (100). Statistical differences between groups were assessed using a two-tailed unpaired Student’s t-test, and a p-value < 0.05 was considered statistically significant.

## Results

### Validation of NAGLU deficiency in reproductive tissues

To confirm NAGLU deficiency in the reproductive tissues examined, NAGLU enzymatic activity was measured in testis (S1A Fig) and epididymis (S1B Fig) of WT and *Naglu-/-* mice. The data showed that there was a higher level of activity in the epididymis than the testis. In either case a significant decrease in activity was noted between WT and KO mice. In addition, an overview of the epididymal epithelium by LM immunofluorescence (LM-IF) demonstrated an accumulation of heparan sulfate throughout the epididymal epithelium in *Naglu-/-* mice (S1C, S1D Fig) suggesting an increase in this substrate.

### Profound alterations of interstitial macrophages in testis of *Naglu*-/- mice

**Fig 1:**
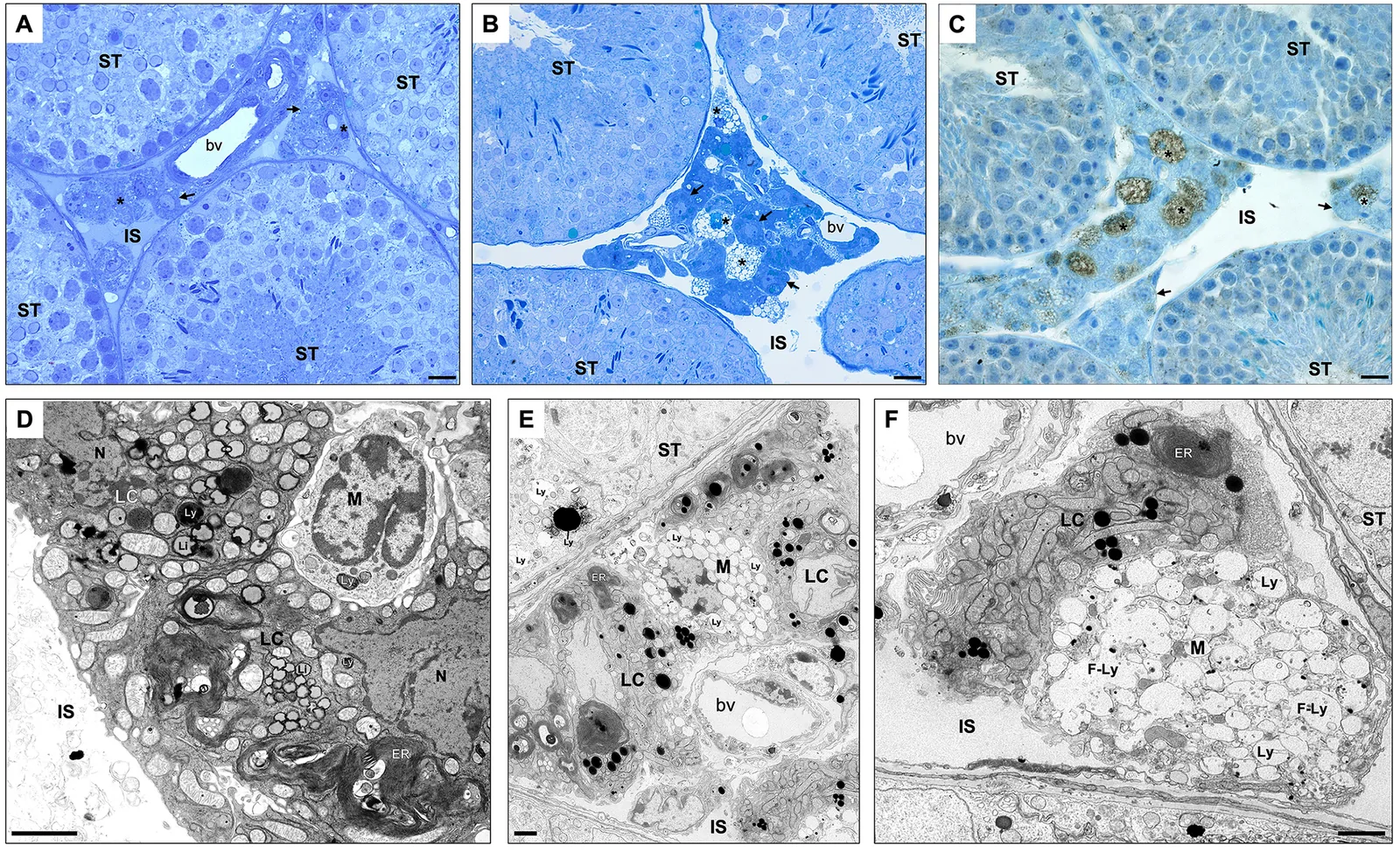
LM toluidine blue-stained sections of testis from WT (A) and *Naglu* KO (B, C) mice and with TEM in WT (D) and KO (E, F) mice. The epithelium of seminiferous tubules (ST) of KO mice (B, C) is comparable to WT mice (A). Macrophages show accumulation of pale lysosomes (asterisks), while Leydig cells (arrows) are similar to WT mice (A, B). In (C), CD68 immunostaining highlights macrophages (asterisks). By TEM (D), WT macrophages (M) display few dense lysosomes (Ly), while in (E, F) accumulations of pale lysosomes some of large size are noted suggesting fusion with one another (F-Ly). In (E, F), Sertoli cells reveal moderate sized dense and empty looking pale lysosomes (Ly). Leydig cells (LC) of WT and KO mice are comparable and contain numerous lipid droplets (Li), dense lysosomes (Ly) and accumulations of smooth endoplasmic reticulum (ER). IS, interstitial space; bv, blood vessel. Scale bars: (A–C)=20 μm; (D–F)=2 μm.

In the testis of *Naglu*-/- mice, at the light microscopic (LM) level, a comparison of the germ cell population at the 12 different stages of the cycle as well as accompanying Sertoli cells did not reveal major differences (Fig 1B and 1C) as compared to wild type mice (Fig 1A). The most profound alteration in the testis occurred in the interstitial space between seminiferous tubules where macrophages exhibited a pale-stained cytoplasm, unlike that of homogeneous moderately dense stained macrophages of WT mice (Fig 1A and 1B). LM immunostaining (LM-IHC) with anti-CD68 antibodies confirmed the identity of the pale-stained cells as macrophages (Fig 1C). By transmission electron microscopy (TEM), spherical macrophages of WT mice displayed few dense lysosomes, while in *Naglu*-/- mice they displayed accumulations of pale lysosomes some of large size. Sertoli cells of KO mice revealed the occasional moderate sized dense or empty looking pale lysosomes, unlike the more compact small spherical dense lysosomes of WT mice. Leydig cells of WT and KO mice were comparable in appearance.

### Absence of profound structural changes of Sertoli and germ cells in testis of *Naglu*-/- mice

Details of Sertoli cell morphology of *Naglu*-/- mice did not reveal any significant structural changes compared to WT mice (compare S2A Fig with S2B-S2D Fig). Notably, in several seminiferous tubules of KO mice, Sertoli cells did display few larger pale-stained lysosomal structures that were localized to their basal cytoplasm and contained loose membranous profiles and electron dense aggregates of varying shapes and sizes (S2B-S2D Fig); these were confirmed to be lysosomal in nature with reactivity for the lysosomal marker prosaposin by LM-IHC (S2C Fig). Such lysosomes did not have a profound impact on the integrity of the seminiferous epithelium. Indeed, the blood testis barrier, Sertoli/germ cell interfaces, germ cell acrosome formation, as well as the developmental sequence of the germ cell populations at all 12 stages of the cycle were not affected. On the other hand, macrophages of *Naglu*-/- mice were filled with numerous pale-stained lysosomes of varying sizes and irregular shapes (S2B Fig and S2D Fig). Myoid cells of KO mice located at the periphery of the tubules in an area referred to as the lamina propria also revealed numerous pale-stained lysosomes (S2D Fig).

### Structural alterations of epithelial cells of the efferent ducts of *Naglu*-/- mice

**Fig 2:**
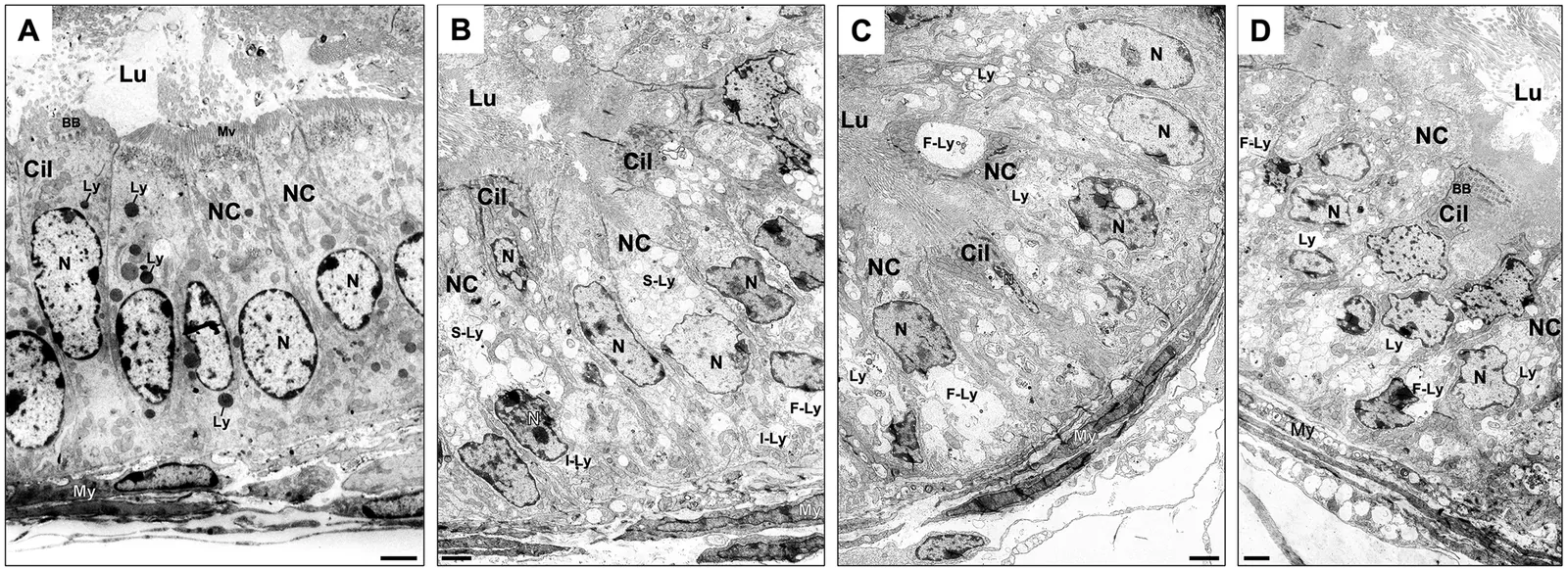
TEM analysis of efferent ducts of WT (A) and KO mice (B-D). In (A), nonciliated cells (NC) display few small dense lysosomes (Ly), a homogeneous microvillar border (Mv) and spherical basally positioned nucleus (N), while ciliated cells (Cil) reveal basal bodies of their cilia (BB) and an elongated more apically located nucleus (N). In (B-D), both cell types demonstrate accumulations of pale-stained lysosomes (Ly) of varying shapes and sizes in their supranuclear (S-Ly, B) and/or infranuclear (I-Ly, B) areas of cytoplasm, with some fusing together (F-Ly, B-D). Myoid cells (My) of the lamina propria contain multiple pale lysosomes (B-D). Lu, lumen. Scale bars = 2 μm.

In *Naglu*-/- mice, both cell types of the efferent ducts, i.e., nonciliated and ciliated cells, were grossly abnormal (Fig 2B-2D) as compared to WT mice (Fig 2A). Overall, the epithelium appeared washed out due to the excessive nature of the accumulated pale-stained lysosomes in the cytoplasm of these cells (Fig 2B-2D). The distinctive feature of nonciliated cells, characterized by their abundant apical microvilli and nucleus positioned more basally, was the preponderance of pale-stained lysosomes of varying shapes and sizes (Fig 2B-2D), some of which appeared to have fused together to form large irregularly shaped structures occupying the supranuclear (Fig 2D) or basal area of their cytoplasm (Fig 2C). Ciliated cells identified by the basal bodies of their cilia and a more apically located nucleus were likewise subjected to a phenotype of numerous pale lysosomes of varying sizes (Fig 2B-2D). Pale stained lysosomes occupied myoid cells of the lamina propria of KO mice (Fig 2B-2D).

### LM analysis of alterations of the epithelium of the epididymis of *Naglu*-/- mice

**Fig 3:**
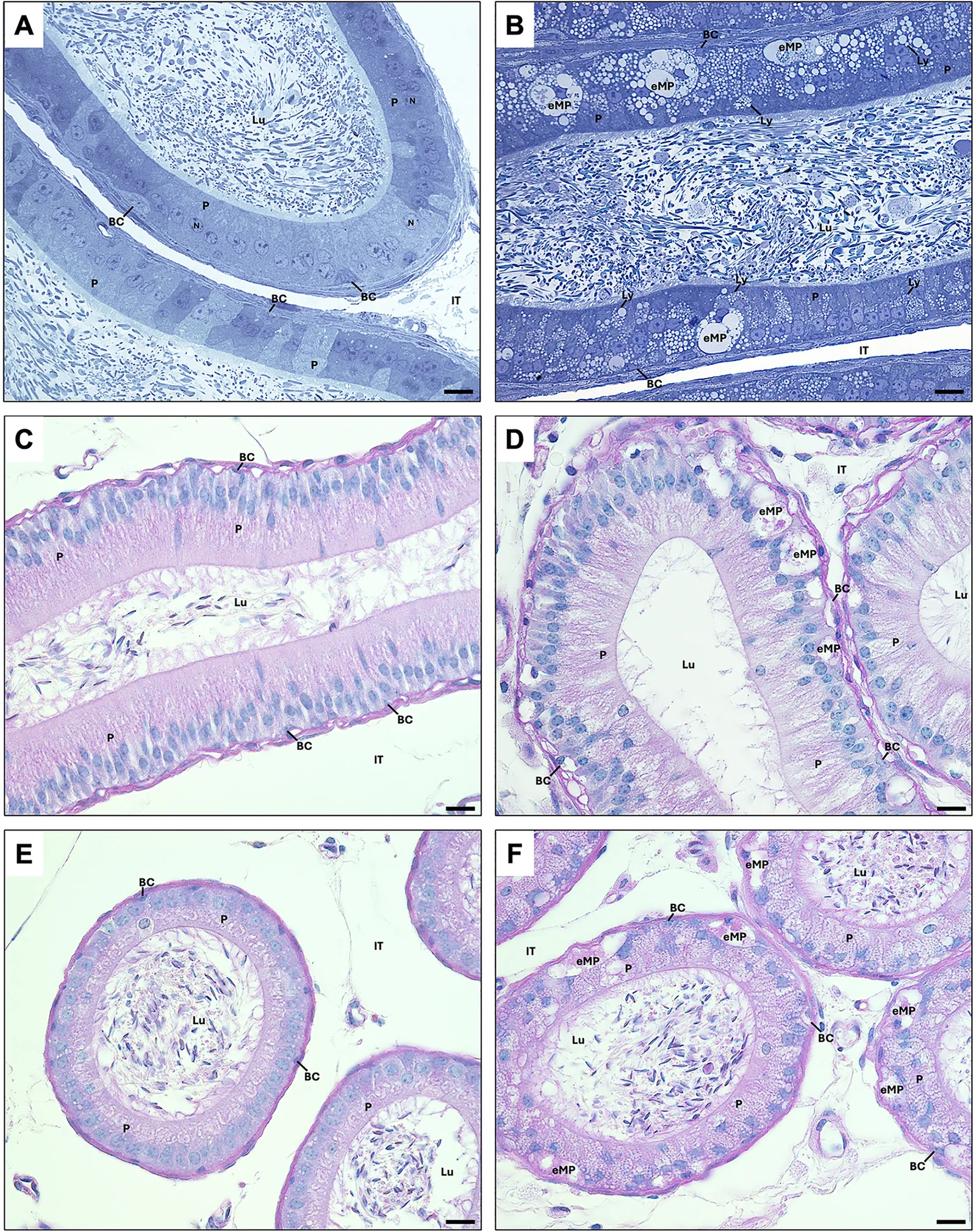
LM toluidine blue-stained sections of the initial segment of the epididymis from WT (A) and *Naglu*-/- (B) mice and PAS-stained sections of WT (C, E) and KO (D, F) mice of the initial segment (C, D) and corpus regions (E, F). In (A, C, E), principal cells (P) demonstrate a pale to moderate-stained appearance and a spherical nucleus (N); small basal cells (BCs) are restricted to the base of the epithelium. In (B, D, F), P cells have a vacuolated supranuclear cytoplasm corresponding to pale stained lysosomes; some BCs contained small vacuoles (B, D, F). Sporadic highly enlarged eMP cells occupy the mid/basal level of the epithelium and present a small nucleus (N) and vacuoles, some of large size (B, D, F); they are not readily discernable in WT mice due to their inactivated state (A, C, E). In (B, F), sperm are abundant in the epididymal lumen. IT, intertubular space; Lu, lumen. Scale bars = 20 μm.

While major changes were noted in all 4 regions of the epididymal duct, our major focus in this study was limited to the initial segment, caput and corpus regions; the cauda is a more complicated study onto itself. LM analysis of the epididymis of WT and *Naglu*-/- mice demonstrated marked alterations in the appearance of epithelial cells of KO mice. In all epididymal regions of *Naglu -/-* mice, principal cells revealed a marked intracellular accumulation of small to medium sized pale-stained lysosomes in their cytoplasm (Fig 3B, 3D and 3F) which greatly contrasted their nondescript appearance in WT mice (Fig 3A, 3C and 3E). Notably, in KO mice, the epithelium demonstrated several enlarged cells with a spherical or highly elongated shape occupying a substantial area of the basal compartment of the epithelium but with no evidence of communication with the lumen and presenting a washed-out appearance containing large empty vacuoles in the company of a small nucleus (Fig 3B, 3D and 3F). Such cells were defined as a population of epididymal mononuclear phagocytes (eMPs). By LM, their appearance in WT mice throughout all epididymal regions was difficult to discern due to their small size, homogeneous staining alongside the other epithelial cells, and absence of a highly vacuolated appearance as noted in KO mice (Fig 3A, 3C and 3E). Basal cells occupied a small territory of the base of the epithelium of all regions; they hugged the basement membrane and in KO mice, some appeared dilated while others did not (Fig 3B, 3D and 3F).

### LM-IHC markers for monocytes/macrophages (M/M), dendritic cells, basal cells and lysosomes of epithelial cells and eMPs of WT and *Naglu*-/- mice

**Fig 4:**
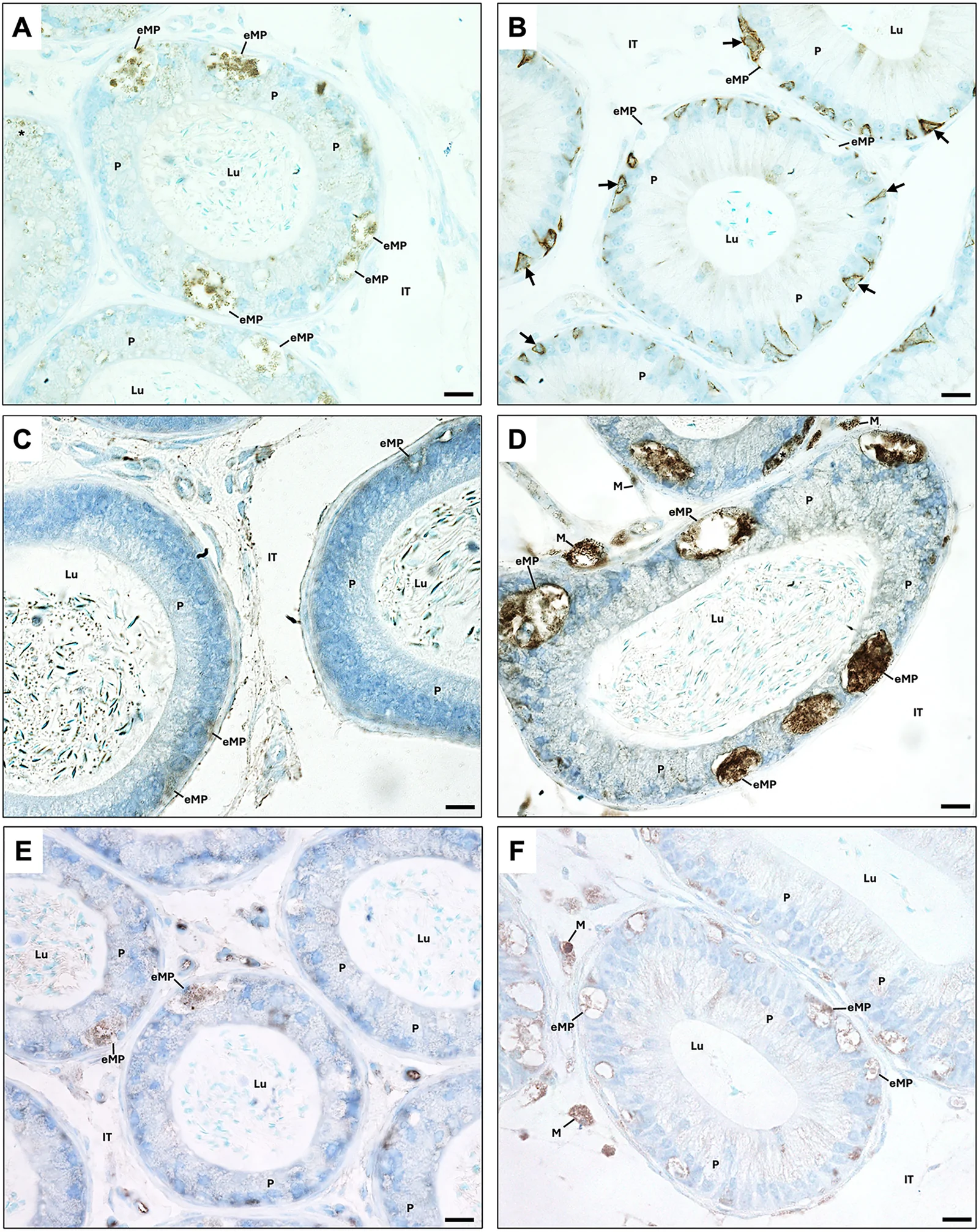
LM immunostaining of the epididymis from WT (C) and *Naglu-/-* (A, B, D–F) mice using anti-PSAP (A, caput), anti-KRT5 (B, initial segment), anti-CD68 (C, D, corpus), anti-CX3CR1 (E, corpus), and anti-MOMA-2 (F, initial segment) antibodies. In (A), P cells reveal a supranuclear punctate reaction with the lysosomal protein PSAP, as do eMPs of KO mice. In (B), basal cells (arrows) are intensely reactive for KRT-5. The eMPs reveal their identity as M/M cells in WT mice (C, CD-68) and KO mice with CD-68 (D) and MOMA-2 (F) antibodies, and in part as dendritic cells with CX3CR1 (E). Intertubular macrophages (M) of KO mice are reactive with the different macrophage markers (D, F). Lu, lumen; IT, intertubular space; Scale bars (A-F)=20μm.

To confirm the identity of the different cell types within the epididymal epithelium of *Naglu*-/- mice, we employed different markers using specific antibodies in conjunction with LM immunochemistry (LM-IHC). In *Naglu*-/- mice (Fig 4A), anti-prosaposin (PSAP) antibody, a marker for lysosomes [49,56] displayed a lysosomal reaction in epithelial cells and eMPs. Cytokeratin-5 (KRT-5) antibody (Fig 4B) revealed a distinct reaction for basal cells, a marker for these cells but not for eMPs [24,57,58]. In WT mice (Fig 4C), some eMPs were reactive for anti-CD68 antibody a macrophage marker [53], however their appearance was enhanced in KO mice due to their highly activated state (Fig 4D). Other eMPs of KO mice were reactive with the dendritic marker CX3CR1 [24] (Fig 4E) and macrophage marker MOMA-2 [55] (Fig 4F). Intertubular macrophages (M) were prominent and enlarged in KO mice and reactive with the different macrophage markers (Fig 4D and 4F).

Additional LM immunofluorescent analysis of lysosomal markers, LAMP2 and cathepsin B, were performed. In WT mice (S3A Fig), clear cells were strongly reactive for LAMP2 due to the accumulation of lysosomes in these highly endocytic cells [59]. Principal cells showed modest punctate reactions for cathepsin B. Interestingly, principal cells of *Naglu-/-* showed a marked increase in reactivity for both markers (compare S3B Fig with WT mice S3A Fig). Moreover, some principal cells demonstrated a marked increase for LAMP2, while others for cathepsin B (S3B Fig and inset Fig). Basal cells of KO mice also showed strong reactivity for LAMP2. On the other hand, eMPs revealed intense cathepsin B immunoreactivity within their enlarged lysosomal compartment and a less prominent reaction for LAMP2 (S3B Fig and inset Fig). In any case, the reactivity for LAMP2 and cathepsin B of KO mice was markedly enhanced compared to WT mice suggesting upregulation of these lysosomal proteins with cell type specificity.

### Low power TEM overview of epididymal epithelium of *Naglu*-/- mice

**Fig 5:**
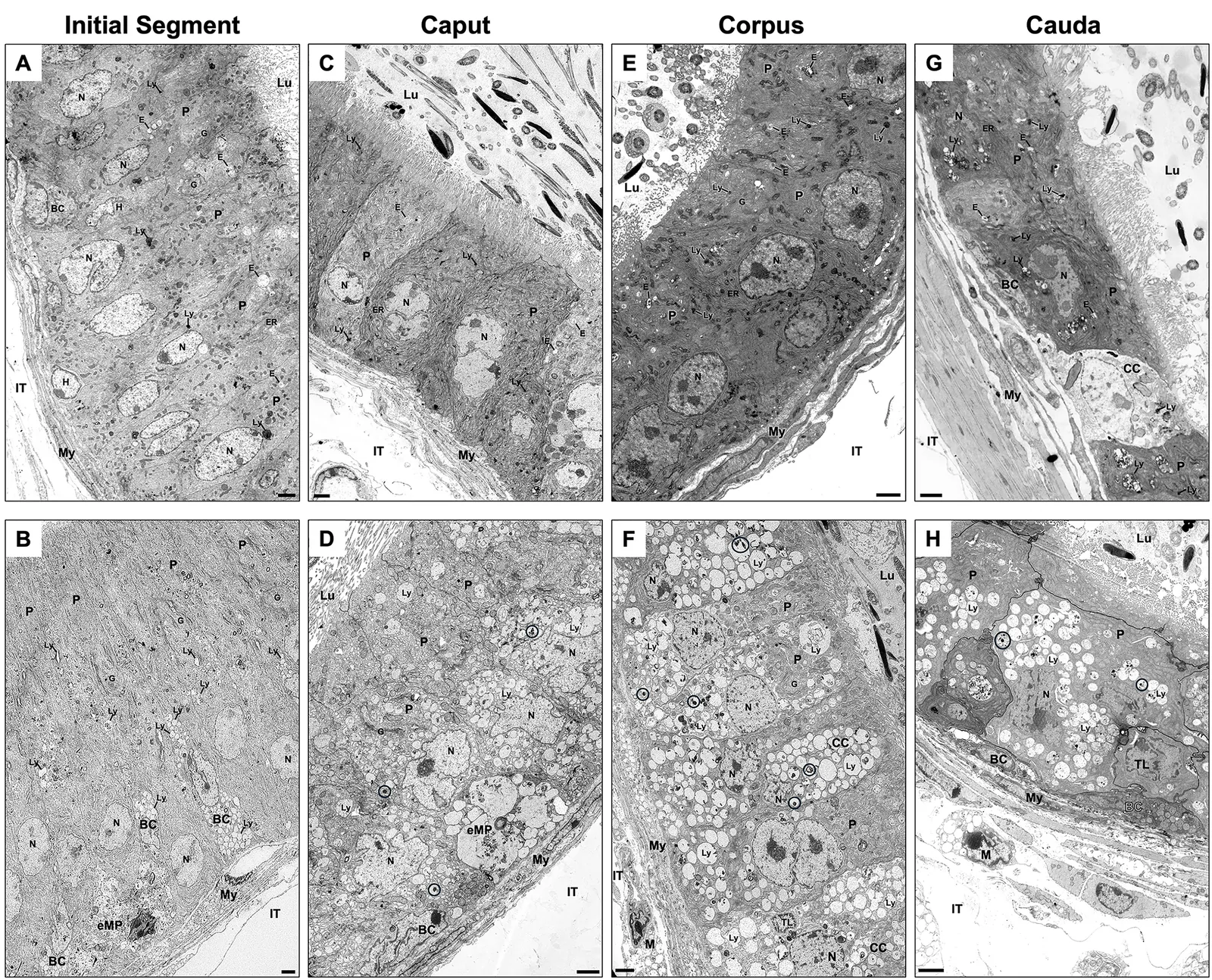
EM of the initial segment (A, B), caput (C, D), corpus (E, F), and cauda (G, H) regions of WT (A, C, E, G) and *Naglu-/-* (B, D, F, H) mice. In (A, C, E, G), P cells present a few dense lysosomes (Ly), endosomes (E), Golgi apparatus (G) and endoplasmic reticulum. In (B, D, F, H), P and clear (CC) cells contain small to moderate-sized, pale to moderately dense lysosomes containing aggregates of electron dense bodies (circles, D, F, H). In (B, D), eMPs display a large, highly vacuolated appearance and occasional nucleus (N). Some BCs of KO mice are filled with small pale lysosomes (B, H), while others are not (D). Myoid cells (My) reveal pale lysosomes (B, D, F), as do intertubular macrophages (F, H). In (F) and (H), T-lymphocytes appear in the epithelium. Lu, lumen; N, nucleus of P cells; H, halo cell. Scale bars = 2 μm.

At low power TEM, the ultrastructural evidence of lysosomal storage pathology was conspicuous throughout the epithelium of all epididymal regions in KO mice. Principal cells of the epididymis of WT mice revealed sparse randomly scattered small dense lysosomes in the cytoplasm (Fig 5A, 5C, 5E and 5G), while in KO mice accumulations of pale lysosomes were noted with varying shapes and sizes and which at times filled the supra- and infra-nuclear cytoplasmic areas (Fig 5B, 5D, 5F and 5H). In KO mice, the nuclei of principal cells were large with a spherical or discoid appearance and a pale chromatin pattern, with some having highly irregular profiles (Fig 5A-5H). Clear cells showed a plethora of supranuclear pale lysosomes. The lysosomes of both principal and clear cells at times contained electron dense granular inclusions of varying sizes and shapes (Fig 5D, 5F and 5H). In KO mice, some basal cells were enlarged and had an orientation towards the apical luminal aspect of the cell and housed pale lysosomes (Fig 5B), whereas others appeared distended, flattened and stretched along the basement membrane displaying pale to moderate stained lysosomes (Fig 5H). Moreover, several basal cells were nondescript and without pale lysosomes (Fig 5D).

In addition, eMPs were noted as large irregularly shaped distended cells prominent in all regions of the epididymis of KO mice, where they were scattered randomly in the basal compartment of the tubule (Fig 5B and 5D). They were filled with large pale lysosomes containing various particulates (Fig 5B and 5D). When present a small dense nucleus appeared to be floating in the interior of the eMP (Fig 5B). Myoid cells of the lamina propria contained pale lysosomes (Fig 5B, 5D and 5F).

### High power TEM of principal cell organelles of the caput and corpus regions of *Naglu*-/- mice

**Fig 6:**
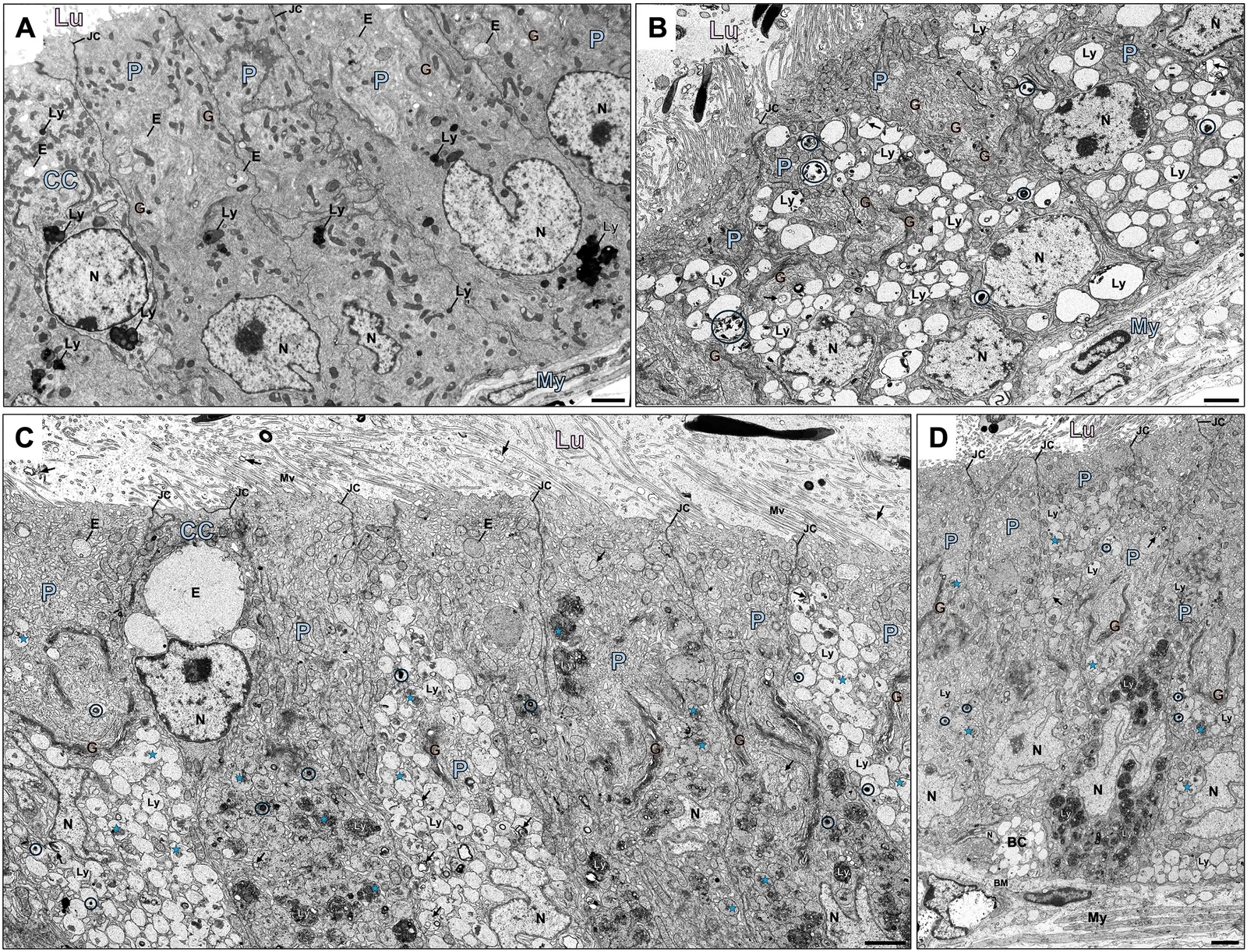
TEM of principal cells of WT (A) and KO (B-D) mice of the initial segment (A), corpus (B) and caput epididymis (C, D). In (A), Golgi apparatus (G), endosomes (E) and small dense lysosomes (Ly) of WT P cells are indicated. In (B-D), P cells display a reduced and loosely arranged Golgi (G) and many lysosomes (Ly). In (B), the lysosomes of P cells ranging from small to large, are translucent and contain sporadic membranous profiles (arrows) and electron dense inclusions (circles). In (C, D), two distinct P cell phenotypes are noted. One phenotype of P cells reveals lysosomes with a moderate to densely stained matrix (labeled with white Ly) containing aggregates of flattened membranous discs (stars), membranous profiles (arrows), electron dense inclusions (circles) and clusters of small dense vesicles. A second P cell phenotype reveals pale stained lysosomes (labeled with dark Ly) with a matrix containing only the occasion membranous disc (stars), membranous profile (arrows), and electron dense inclusion (circles). In (C), the membranous profiles of epididymal lumen are indicated (arrows). JC, junctional complex; BC, basal cell; N, nuclei of principal cells; CC, clear cell; E, endosome; My, myoid cell. Scale bars = 2 μm.

In WT mice, principal cells revealed a supranuclear Golgi apparatus consisting of several elaborate stacks of flattened intertwined saccules (Fig 6A). Endosomes were noted apically, while several small densely stained lysosomes with a spherical or irregular shape were noted next to the Golgi apparatus; a large spherical nucleus was noted basally (Fig 6A). Clear cells contained few dense lysosomes and a mid-epithelial position of their nucleus (Fig 6A). In principal cells of KO mice (Fig 6B-6D), the Golgi revealed fewer stacks of saccules (Fig 6B-6D). While nuclei of some principal cells had spherical or discoid shapes and with few surface indentations (Fig 6B), others were lobulated and irregular in form (Fig 6C and 6D). The presence of closely apposed tight junctional complexes at the apical front of the epithelium between adjacent principal cells suggested an intact and functional blood epididymal barrier (Fig 6B-6D).

However, the most conspicuous abnormality in principal cells of KO mice was the plethora of pale to moderately stained lysosomes of differing shapes, sizes and content that included a cell- and region-specific phenomenon (Fig 6B-6D). In the initial segment of KO mice, abnormal pale lysosomes were evident in principal cells but not overpowering the cells (Fig 5B). In the corpus (Fig 6B) principal cells revealed a homogeneous population of lysosomes that were small to large and predominantly translucent, containing few loosely assembled membranous profiles and whorls or plaques of electron dense material of varying shapes and sizes.

In contrast, in the caput region, two distinct principal cell phenotypes were noted based on their lysosomal population; those bearing lysosomes with a pale-stained appearance versus those with a moderate to densely stained matrix (Fig 6C and 6D and S4A and S4B Fig). In the case of the latter (Fig 6C and 6D and S4 Fig blue boxes), their content revealed a matrix packed with aggregates of small closely aligned parallel flattened compacted membranous discs as noted in side views and with a fingerprint-like impression in face views, in addition to clusters of small densely stained vesicular profiles. On the other hand, lysosomes of the second principal cell phenotype (S4 Fig purple boxes) had a consistently pale stained matrix containing only the occasion aggregate of membranous discs with fingerprint impressions. Furthermore, various images of principal cell lysosomes of the caput region indicated signs of intimate contact and fusion; the latter appeared as large, elongated, distended lysosomes with a wavy, irregular profile or as conjoined spherical bodies (Fig 6C and 6D and S4 Fig blue and purple boxes).

Another feature noted in both populations of principal cells of the caput was the presence of abnormal mitochondria, a finding not observed in other regions. Such images of mitochondria in the cytoplasm suggested initiation of atrophy (S4 Fig red boxes: S4A1-S4A4 Fig and S4B1-S4B3 Fig). These included a spherical mitochondria-like body surrounding a central pale space (S4A1 Fig red box); a similar structure appearing with a moderately stained lysosome (S4A2 Fig red box); a partially intact mitochondria appearing to have fused with a moderately stained lysosome (S4A3 Fig red box); and mitochondria with a more intact shape but revealing a moderate sized pale-stained area between cristae (S4A4 Fig red box). Some abnormal mitochondria appeared to have fused with a moderately stained lysosome or were engulfed by a lysosome and lodged within its matrix (S4B1 and S4B3 Fig red boxes). Lysosomes associated with such mitochondria displayed fingerlike impressions (S4B1 and S4B2 Fig red boxes).

### TEM analysis of structural alterations of eMPs of *Naglu*-/- mice

**Fig 7:**
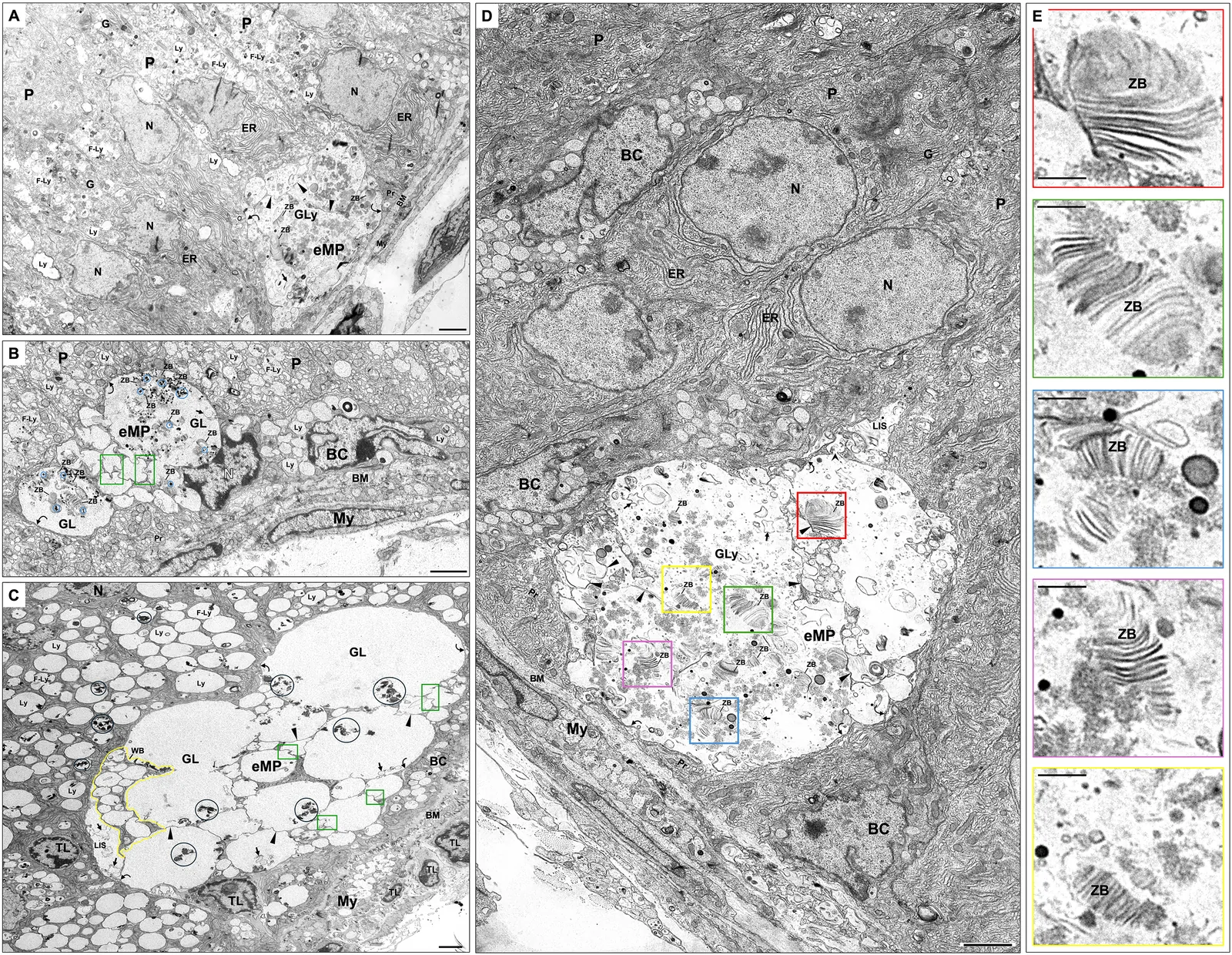
TEM of eMPs sporadically distributed in the basal compartment of the epithelium of KO mice of the initial segment (A, D), caput (B) and corpus C). In (A-D), eMPs are dominated by pale-stained lysosomes (Ly) ranging from small (Ly) to giant (GL) (B, C) and gigantic (Gly) (A, D); small digit like projections of adjacent lysosomes poke into one another (green boxes). The cytoplasmic matrix forming the peripheral rim (curved arrows) and thin filamentous projections (large arrowheads) extending inward and enveloping the numerous pale lysosomes are indicated (A-D); a web-like network is outlined in yellow (C). In (D), the peripheral rim is broken (indented arrowhead) with release of lysosomal contents into the lateral intercellular space (LIS). eMPs contain short flattened membranous zebra bodies (ZB, A, B, D, E insets). In (C), small membranous profiles (arrows) and electron dense plaques (circles) appear in eMPs similar to those seen in the lysosomes of P cells. Some eMPs are separated from the basement membrane (BM) via by principal cell cytoplasm or processes (Pr) (A-C), while others are in direct contact (D). My, myoid cells; F-Ly, fused lysosomes; G, Golgi; ER, endoplasmic reticulum; BM: basement membrane; N, nucleus, BC, basal cells; TL, T-lymphocytes. Scale bars = 2 μm; Inset scale bars = 500 nm.

**Fig 8:**
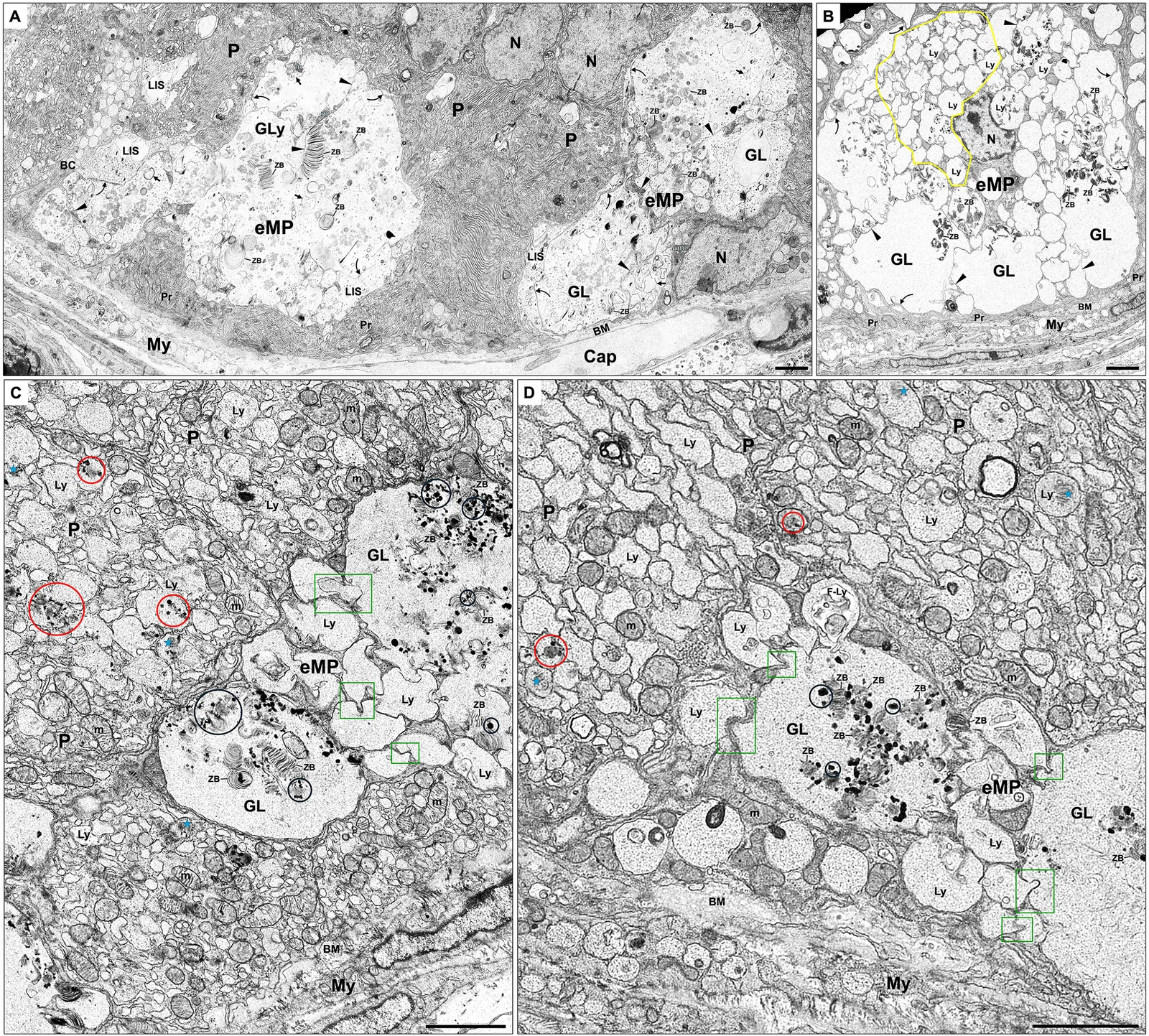
TEM features of eMPs of KO mice of the initial segment (A), corpus (B) and caput (C, D) epididymis. In (A-D), enlarged eMPs are present, containing giant (GL) and gigantic (GLy) lysosomes. In (A-C), the thin peripheral rim (curved arrows), filamentous projections (arrowheads) of the cytoplasmic matrix and web-like network (area enclosed by yellow circle) of an eMP are indicated. The eMPs are either in direct contact with the basement membrane (BM) (A, D) or separated from it (A-C). In (A, B), the nucleus (N) and odd mitochondria (m) occupy an eMP and in (A), a thin extension of the dilated area (arrowhead) separates the 2 Gly from each other. In (A), the peripheral rim of the eMP is disrupted releasing lysosomal loose membranous profiles (arrows) into the lateral intercellular space (LIS). In (C, D), the GL of eMPs contains zebra bodies (ZB) associated with small electron dense bodies (dark circles) which are also noted in lysosomes of P cells (red circles) along with small disc-like fingerprint impressions (stars). Green boxes, small digit-like processes; F-Ly, fused lysosomes; Scale bars = 2 μm.

In this part of our study, the major focus was to examine the ultrastructural features of eMPs from the initial segment to the distal corpus regions, as the cauda region presented a more complex phenotype involving the epithelium, lamina propria and intertubular spaces. The eMPs of *Naglu-/-* mice presented themselves as large irregularly shaped vacuolated cells that occupied a large area of the basal compartment of the epithelium and with a sporadic appearance along the epididymal duct (Fig 7A-7D; Fig 8A and 8B). The eMPs were characterized by the presence of pale lysosomes of varying sizes and highly irregular forms (Fig 7A-7D; Fig 8A and 8B).

For the most part, eMPs were large in size and dominated by a single gigantic membrane bound lysosome that occupied virtually the entire cytoplasmic matrix of the cell (Fig 7A, 7C and 7D; Fig 8A and 8B), while other eMPs were occupied by pale lysosomes of varying shapes and sizes from small to giant (Fig 7B; Fig 8A and 8B). Remarkably, closer inspection of eMPs revealed that their cytoplasm was reduced in size due to constriction by numerous pale lysosomes of varying sizes, some of gigantic size, which profoundly impacted the appearance and overall integrity of these cells (Fig 7A-7D; Fig 8A and 8B). Due to the abundance and size of the pale lysosomes, the cytoplasmic matrix and presence of typical organelles of the eMPs were severely impacted. At their outermost edge, the cytoplasmic matrix was constricted where it appeared as a thin densely stained thread-like rim of cytoplasm, i.e., peripheral rim. From the outermost edge of the eMP, the peripheral rim of cytoplasmic matrix was seen to extend inwards, as thin filamentous-like threads, deep into the interior of the cell where they intimately enveloped each pale lysosome that they encountered (Fig 7A-7D; Fig 8B). The thin “peripheral rim” was composed of the opposing plasma membranes outlining on the one hand the outer plasma membrane of the eMP and on the other hand that of the gigantic lysosome or numerous pale lysosomes of varying sizes situated close to the outer edge of the eMP. The area enclosed by the opposing membranes represented the cytoplasmic matrix and consisted solely of a thin layer of homogeneous electron dense. As the thin “filamentous-like” projections of the cytoplasmic matrix extended deep into the cell to envelop the various lysosomes encountered, they formed at times sporadic indentations in the larger pale lysosomes, resulting in their highly irregular forms. At times, the filamentous-like matrix encompassing the pale lysosomes formed a web-like network (Fig 7A-7D; Fig 8A and 8B). Consequently, the integrity of the cytoplasm was squished into its abnormal shape by the presence of all the numerous pale lysosomes of different shapes and sizes.

Notably, the nucleus of eMPs was not conspicuous and only in rare planes of section was it evident. When present, the filamentous lysosome loaded cytoplasmic web-like network was expanded and caused significant indentation of its profile (Fig 7B and Fig 8B). At these dilated areas of cytoplasm and elsewhere, the absence of typical organelles, e.g., Golgi apparatus, ER cisternae and cytoskeletal elements, was noticeable, and only on occasion were small mitochondria located in the filamentous cytoplasmic network (Fig 8B).

An added feature of all eMPs in *Naglu*-/- mice was the presence of small well-defined clusters of closely stacked short, flattened, parallel membranous discs referred to as zebra bodies within their pale lysosomes (Fig 7A, B, D, E; Fig 8-D and S5A-S5D Fig). These discs in side views appeared as small individual distinct membranous profiles aligned in parallel, with a moderately stained appearance and having a straight or wavy appearance, while in surface views they appeared diffuse, blurry, and plate like (Fig 7A, 7D and 7E, insets; Fig 8A, 8C and 8D). The discs of a given zebra body were often closely associated with similarly oriented discs parallel to one edge of the cluster (Fig 7D and 7E, insets; Fig 8A). The association at these sites resembled the pages of a book attached to its binding. Zebra bodies were either cylindrical, curved or circular in shape (Fig 7A-7E, insets; Fig 8A-8D). Curiously another prominent feature of the interior of the enlarged lysosomal compartments of eMPs was the presence of numerous electron dense bodies of spherical shape and small size that formed distinct patches integrated with zebra bodies (Fig 7B; Fig 8C and 8D). Taken together, the finding of zebra bodies and electron dense bodies suggests a phagocytic activity for the eMPs and their segregation with their enlarged lysosomal compartments.

A detailed TEM comparison of the membranous discs forming zebra bodies of eMPs and that of the cristae of mitochondria of principal cells revealed a remarkable similarity between the two (Fig 7E insets; Fig 8C and 8D).

To further characterize the zebra bodies accumulated within the enlarged lysosomal compartments of eMPs, SCMAS immunostaining was performed on epididymal sections (S6 Fig). SCMAS (subunit c of mitochondrial ATP synthase) immunoreactivity was detected in epithelial cells but notably in structures within the enlarged lysosomal compartments of eMPs (S6A Fig and inset Fig). The detection of SCMAS immunoreactivity within enlarged lysosomal compartments of eMPs provides additional evidence for the accumulation of mitochondrial-derived material within these structures but future TEM immunolocalization would be more conclusive.

Added features of eMPs included the findings that on occasion, the outer plasma membrane and peripheral rim appeared to be fragmented at points along its length (Fig 7D). At these rupture sites, the contents of the pale lysosomes appeared to be released into the lateral intercellular space. Indeed, the presence of loose membranous profiles, small vesicular elements and fine particulate material were noted both in the lateral intercellular spaces as well as the interior of the giant pale lysosomes of eMPs suggesting their release from the latter during their fragmentation process (Fig 7C and 7D; Fig 8A). Such findings suggested that some eMPs were undergoing degeneration possibly due to their congested state.

### Different cell populations of eMPs exist in the epididymis of *Naglu*-/- mice with regard to associations with the basement membrane and with T-lymphocytes

In the present study, we noted that some M/M cells of the eMP family occupied a mid-epithelial position and as such were well removed from the basement membrane suggesting a circulating M/M identity (Fig 7A-7D; 8A-8D and S5A Fig), as defined for a subpopulation of halo cells in the early literature [8,13,20]. In such cases, the M/M cells were separated from the basement membrane by large areas of the basal cytoplasm of principal cells (Fig 7A and 7B; Fig 8A-8C; S5A Fig) or long thin processes of basal cells (Fig 7A-7C; Fig 8A and 8B). On the other hand, other M/M cells had an intimate association with the basement membrane (Fig 7A; 8A and 8D; S5A-S5D Fig), suggesting a resident M/M identity.

Of added interest was the presence of T-lymphocytes associated with some eMPs (Fig 7C) suggestive of a dendritic nature for these cells, as defined in the literature [60]. The T-lymphocytes did not present any lysosomal abnormalities and resembled those of WT mice.

### M/M cells of the intertubular space of the epididymis of *Naglu*-/- mice

In addition to M/M cells within the epididymal epithelium, examination of the intertubular space of the epididymis by TEM revealed the presence of M/M cells with an altered phenotype in KO mice. Such cells contained a small nucleus and an abundance of pale lysosomes of varying sizes that occupied most of the cytoplasm (S7B and S7C Fig), compared to the few small sporadic dense lysosomes of WT mice (S7A Fig). Interestingly, none of the lysosomes of intertubular macrophages of the epididymis of KO mice exhibited zebra bodies, and this was also the case for macrophages of the testicular interstitial space (Fig 1 and S3 Fig). These findings suggest different substrates acted upon by macrophages in the different compartments of the testicular and epididymal milieu.

### Ultrastructural alteration of epididymal epithelium suggesting structural abnormalities of principal cells

**Fig 9:**
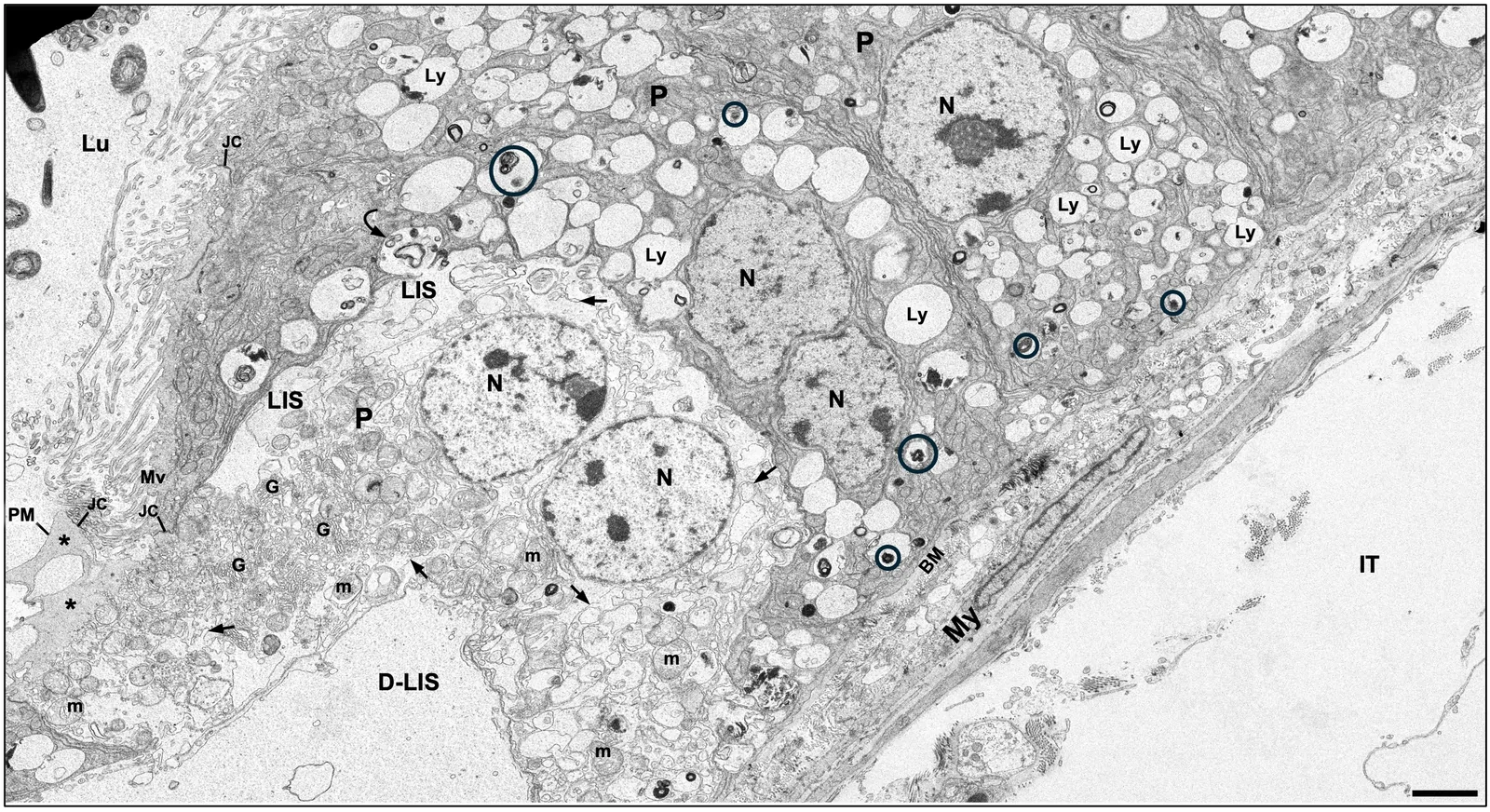
An abnormal principal cell (P) of the corpus region showing disrupted lateral plasma membrane, two nuclei (N), a disorganized Golgi apparatus (G), bloated mitochondria (m) and numerous isolated empty-looking membranous profiles (arrows). The lateral intercellular space (LIS) contains organelles and membranous profiles and bulges into an adjacent principal cell (curved arrow). The apical plasma membrane (PM) lacks microvilli but is intact, beneath which the cytoplasm contains finely granular material lacking organelles (asterisks). Junctional complexes (JC) of this cell are retained with adjacent cells. The adjacent principal cell (P) houses two nuclei and a cytoplasm filled with pale lysosomes (Ly) containing an electron dense material (circles). Mv, microvilli; D-LIS, dilated intercellular space; BM, basement membrane, My, myoid cell, IT, intertubular space. Scale bars = 2 μm.

Observations made in the epididymal epithelium suggested that few principal cells were undergoing structural alterations suggestive of degeneration to their normal state (Fig 9). Such abnormal cells displayed several nuclei, a Golgi apparatus seen as small, disorganized stacks, and bloated mitochondria; the absence of pale lysosomes was notable. In addition, numerous empty-looking membranous profiles of varying shapes and sizes were noted in the cytoplasm but without identification. Notably, the lateral plasma membrane of the abnormal cell opposing the adjacent principal cell of the epithelium appeared to be disrupted whereupon its organelles and membranous profiles floated in the lateral intercellular space. A large highly dilated intercellular space of the epithelium faced the lateral plasma membrane of the abnormal principal cell (Fig 9).

**Fig 10:**
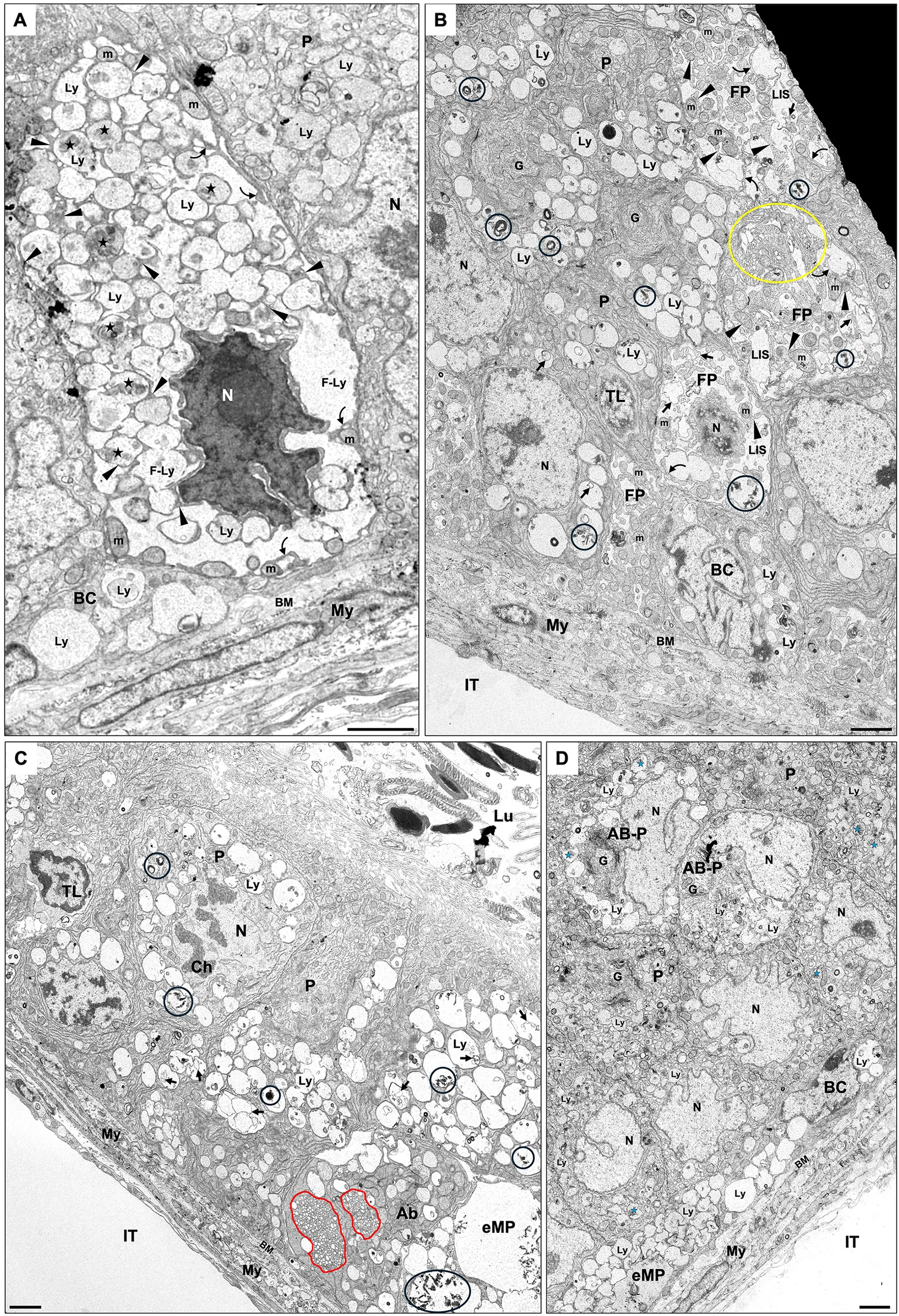
TEM of abnormal principal cells (P) of KO mice of the caput (A, D) and corpus (B, C) epididymis. In (A and B), abnormal P cells display an abnormal cytoplasmic matrix, formed as a thin peripheral rim (curved arrows) and inner filamentous-like (arrowheads) (arrowheads) and web-like network yellow circle), all of which envelop pale lysosomes (Ly) containing few mitochondria. Fingerprint-like impressions (stars) are noted in lysosomes of P cells typical of the caput region. In (B), the lateral intercellular spaces (LIS) contain membranous profiles (arrows) and electron dense bodies (black circles). Pyknotic nuclei (N) appear in P cells. In (C), an abnormal P cell reveals condensed mitotic chromosomes (Ch) in its nucleus (N). A basally lodged eMP contains electron dense material as noted in pale lysosomes of adjacent P cells (black circles). An abnormal mass of cytoplasm (Ab) of unidentified nature, situated next to an eMP, contains pale lysosomes and aggregates of small vesicular profiles (red circles). In (D), two cross sectional profiles of abnormal P cells (Ab-P) present irregular nuclear profiles and a mid-epithelial position; they reveal a Golgi apparatus (G) and pale lysosomes (Ly). The basally located nuclei (N) of adjacent P cells present nuclei with irregular and pale stained profiles. In (A, B and D), a BC adheres to the BM and contains pale lysosomes (Ly). In (C) and (D), myoid cells (My) are loaded with pale lysosomes. In (A-C), T-lymphocytes (TL) are noted in the epithelium. F-Ly, fused lysosomes; Scale bars = 2 μm.

In addition, other TEM images suggested additional pronounced morphological abnormalities indicative of degeneration affecting principal cells. In such cases, principal cells appeared to lose contact with the lumen and basement membrane. In particular, the nucleus was highly irregular in outline and contained densely packed flakes of chromatin, suggestive of pyknosis (Fig 10A). In addition, the cytoplasm was nondistinctive and lacked characteristic organelles, such as Golgi and ER. In fact, the cytoplasm was reduced to a thin peripheral rim with dilations along its length, housing occasional small mitochondria, and was continuous with a conspicuous web-like network of the cytoplasm that enveloped small to moderate sized pale lysosomes (Fig 10A). The small pale lysosomes dominated the existing cytoplasm of the degenerating cell, with some fusing together. Fingerprint-like impressions noted in lysosomes of these cells were typical of those noted in principal cells of the caput region (Fig 10A).

At times, fragmented portions of an altered principal cell were noted spanning the epithelium from the basement membrane to the lumen in a discontinuous manner (Fig 10B). Each fragment of the principal cell was formed of thin finger-like remnants of the cytoplasmic matrix seen floating freely in the lateral intercellular spaces. A small, abnormal nucleus with few patches of densely packed chromatin were noted in a dilated area of the cytoplasmic matrix (Fig 10B). In other compartments of the fragmented principal cell, the web-like network of the cytoplasmic network was partially maintained (Fig 10B). Of notable interest was the absence of well characterized pale lysosomes in the cytoplasmic matrix of these fragments, as well as recognizable organelles. Moreover, the presence of sporadic loosely assembled membranous profiles and whorls or plaques of electron dense material with spherical or irregular outlines, constituents of the pale lysosomes of adjacent intact principal cells, was noted in the lateral intercellular spaces (Fig 10B). This suggested that upon altercation of the principal cell, lysosomes underwent disintegration, liberating their contents into these lateral spaces. The only noticeable organelle of the fragmented remnants were occasional small mitochondria (Fig 10B).

In other cases, principal cells appeared to be displaced and occupied a more central area of the epithelium and without apparent contact with the basement membrane (Fig 10C and 10D), with the nucleus presenting few irregularly shaped chromatin profiles suggestive of mitosis (Fig 10C). A basally lodged eMP contained whorls and plaques of electron dense material with spherical or irregular outlines as noted in the numerous pale lysosomes of adjacent principal cells (Fig 10C). Other cross-sectional profiles of abnormal principal cells with a mid-epithelial position revealed a highly irregular lobulated nuclear profile with a pale stained appearance, a small Golgi apparatus but conspicuous pale lysosomes (Fig 10D). Notably, the adjacent principal cells maintained a full columnar appearance but presented similar irregular nuclear profiles (Fig 10D). Taken together such observations suggested that a small percentage of principal cells of KO mice presented abnormal morphological features suggestive of degeneration/fragmentation.

To determine whether epithelial cells were undergoing degeneration, TUNEL immunostaining was performed on epididymal sections (S8 Fig). TUNEL labeling was markedly increased in *Naglu-/-* (S8B, S8C Fig and inset Fig) compared with WT (S8A Fig) animals. However, particularly strong immunostaining was observed within the large lysosomal compartments of eMPs. TUNEL-positive labeling was also observed in the epithelial compartment, represented as a punctate cytoplasmic reaction in principal cells, as well as a strong reaction for the occasional basal cell.

Interestingly, the nuclei of eMPs, when present in the plane of section, appeared intact and were not TUNEL-positive, whereas TUNEL-positive material was observed within their enlarged lysosomal compartments, associated with DAPI-positive material (S8C Fig and inset Fig). The presence of TUNEL-positive material within the enlarged lysosomal compartments of eMPs may correlate with the presence of patches of electron dense bodies often of spherical shape noted within the interior of the enlarged lysosomal compartment of eMPs. These distinct patches may correspond to fragmented DNA of epithelial cells phagocytosed by eMPS, well recognized as highly effective phagocytic cells.

### Ultrastructural appearance of basal cells of WT and *Naglu*-/- mice

**Fig 11:**
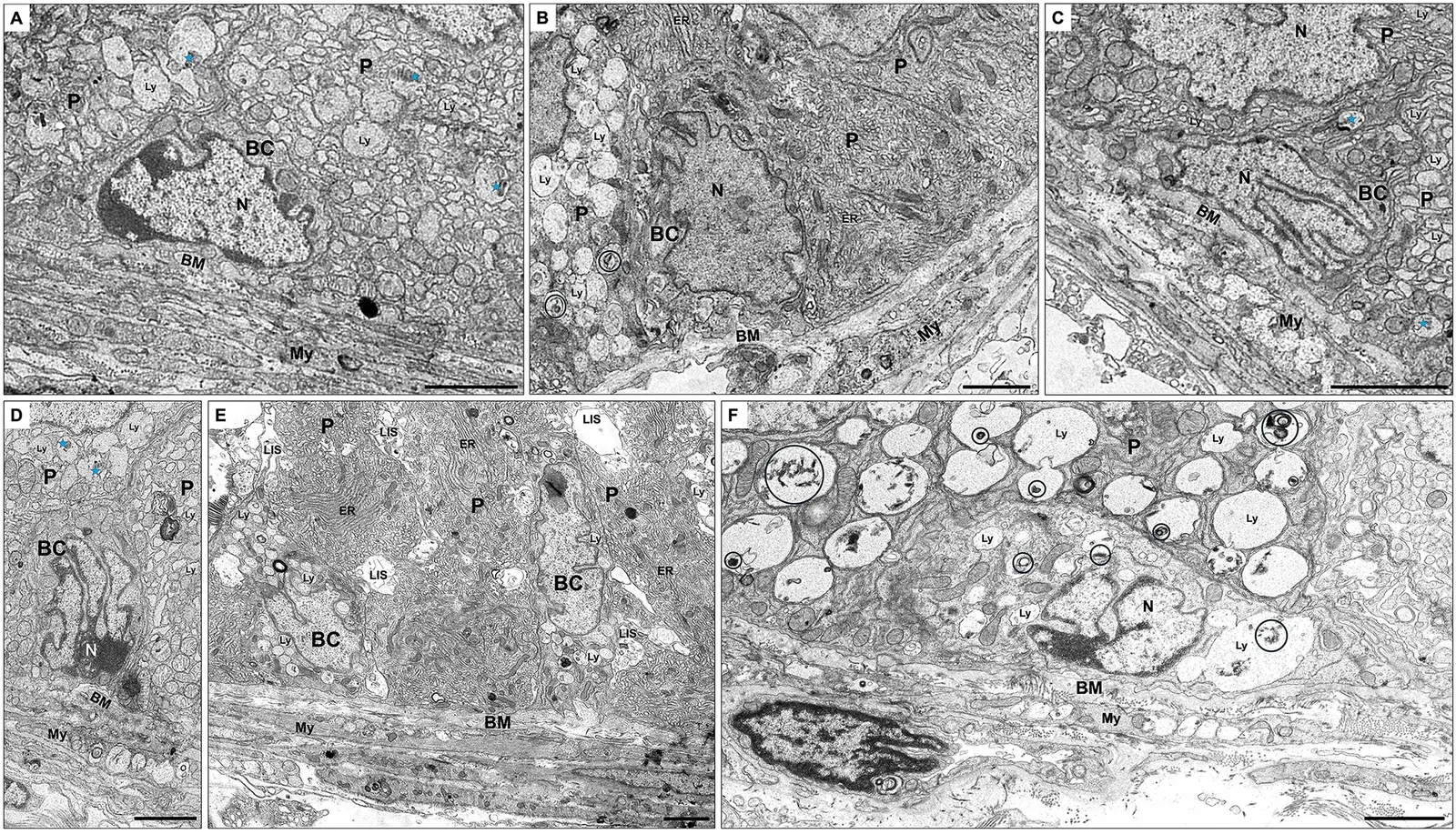
Basal cells (BCs) of KO mice from the caput (A, C, D), initial segment (B, E) and corpus (F) epididymis. In (A-D), BCs present a primitive stem cell-like appearance and contact the BM. In (A) and (B), the BC nucleus (N) is round or oblong with few small indentations, while in (C) and (D) it is highly lobulated and irregular. In (E), the BC is enlarged and orients toward the lumen, while in (F), it is dilated and stretches along the BM housing many pale lysosomes (Ly). In (A, D), lysosomes of P cells exhibit small disc-like fingerprint impressions (stars). In (B, F), a P cell presents pale lysosomes with electron dense whorls (circles), as also seen in a BC. My, myoid cells; ER, endoplasmic reticulum; LIS, lateral intercellular space; circles. Scale bars = 2 μm.

Unlike the large irregularly shaped eMPs of KO mice, dominated by pale lysosomes of varying sizes, basal cells were smaller in size and did not display large or giant pale lysosomes. and had different orientations within the epithelium. Some enlarged basal cells had their main cytoplasmic mass oriented apically towards the lumen; they were filled with pale lysosomes of small to moderate size (Fig 5B; Fig 8D and Fig 11E), while others bearing numerous pale lysosomes were dilated but had an elongated shape, with a circumferential orientation along the basement membrane (Fig 5H; Fig 9A, 9B and 9D; 11F and S7C Fig). Moreover, some basal cells in KO mice did not display any structural abnormalities (Fig 5D; Fig 7D; Fig 11A-11D). Apart from the differences in size and shape of basal cells, the nuclei of basal cells of KO mice had variable features; some were round with small surface indentations (Fig 11A and 11B), while others were slightly enlarged and irregular in form, with deep surface indentations (Fig 11C-11F and S7C Fig). All basal cells had a firm commitment with the basement membrane (Fig 11A-F) and never exhibited zebra bodies.

### Luminal contents of the epididymis of *Naglu*-/- mice

**Fig 12:**
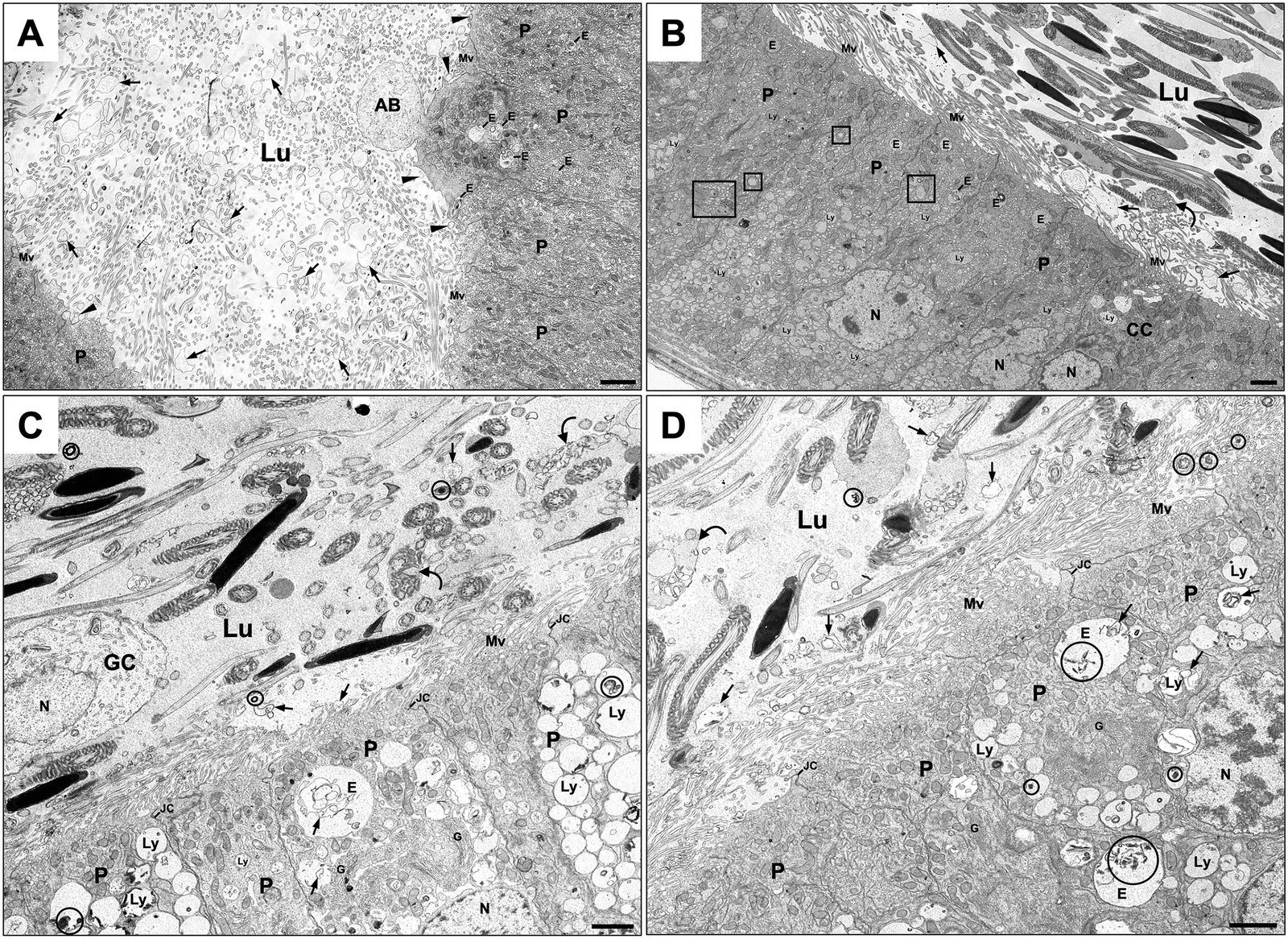
EM of luminal contents of epididymis of the initial segment (A), caput (B) and corpus (C, D) regions of KO mice. In (A-D), numerous loose membranous profiles (arrows) and electron dense material (circles) are scattered in the epididymal lumen (Lu), amongst the microvilli (Mv), near the apical cell surface (arrowheads) and in apically located endosomes (E) and pale lysosomes (Ly) of P cells. In the caput region (B), P cells contain moderately dense lysosomes (Ly) and basally positioned nuclei (N), while a clear cell (CC) has few apically pale stained lysosomes (Ly) and a more centrally located nucleus (N). In (B-D), clusters of small vesicular elements of unidentified nature are indicated (curved arrows). JC, junctional complex; G, Golgi apparatus; GC, germ cell; AB, apical bleb; abnormal mitochondria (boxed in). Scale bars = 2 μm.

A survey of the lumen of the epididymis of KO mice demonstrated several abnormal structures in addition to spermatozoa (Fig 12A-12D). In particular, numerous loose empty-looking membranous vesicular profiles of varying sizes and shapes were randomly scattered in the epididymal lumen, with some approaching the cell surface of the epithelium (Fig 12A-12D). Whorls of highly osmiophilic material and small spherical electron dense aggregates were also evident, in addition to clusters of small vesicular elements of unidentified nature (Fig 12A-12D). Apically located endosomes (E) of principal cells displayed membranous profiles and electron dense whorls or aggregates (Fig 12A-12D). While principal cells of the caput region contained numerous small sized, moderately dense lysosomes (Fig 12A and 12B), those of the corpus region displayed moderate and large sized pale lysosomes containing membranous profiles and electron dense aggregates (Fig 12C and 12D), suggesting their internalization from the lumen.

## Discussion

### *Naglu* gene inactivation reveals ultrastructural alterations to interstitial macrophages of the testis

An overview of the seminiferous epithelium of the testis of *Naglu -/-* mice did not reveal any significant changes to Sertoli cells as compared to WT mice. The finding of a normal phenotype for germ cells of the seminiferous epithelium was substantiated by the abundance of sperm in the epididymal lumen of KO mice. Interestingly, the mild effect on the seminiferous epithelium of *Naglu-/-* mice directly contrasted with its abnormal appearance in *Hgsnat-/-* mice [49], a rationale requiring future investigation.

Conversely, macrophages in the interstitial space, a normal component of WT mice [61,62] were highly compromised in KO mice and contained multiple large pale lysosomes. Macrophages are an essential component of the interstitial space where they serve an immunological protective role [6,63]. In the cathepsin A (PPCA-/-) mouse model, there was a dramatic increase of macrophages with pale lysosomes in the interstitial space, suggesting important immune functions for these cells in both wild type and PPCA -/- mice [64]. Our findings of the present study suggest that macrophages in a normal state endocytose HSPGs but in the absence of NAGLU are unable to degrade it, resulting in the characteristic appearance of a lysosomal storage disease.

### *Naglu* gene inactivation reveals ultrastructural alterations to nonciliated and ciliated epithelial cells of the efferent ductules

In the efferent ducts of rodents (6–20 small ducts), the resident nonciliated and ciliated cells [65,66], demonstrated a plethora of pale-stained lysosomes that disrupted the appearance and distribution of their organelles such as Golgi and ER. In WT mice, nonciliated cells are characterized by a well-developed endocytic apparatus and express key ion channels and transporters that are crucial for maintaining luminal fluid homeostasis and ductule morphology under the control of estrogen signaling [7,65,67–69]. Ciliated cells possess a more modest endocytic apparatus [70] and motile cilia that generate dynamic movements to propel luminal sperm [71,72]. Nevertheless, the structural abnormalities of the efferent ductules of *Naglu-/-* mice did not have serious consequences on fluid accumulation in the lumen to the epididymis. Taken together the data suggests an active role for uptake of HSPGs by epithelial cells of the efferent ductules in KO mice and a great degree of resilience under due stress to maintain their essential roles.

### *Naglu* gene inactivation reveals ultrastructural alterations to principal cells of the epididymis

In the present study, an analysis of *Naglu*-/- mice, performed mainly from the initial segment to the distal corpus epididymis, demonstrated major structural alterations to few principal cells of the epididymis.

In *Naglu-/-* mice, the cytoplasm of principal cells contained multiple pale lysosomes that often filled their entire cytoplasm, with some of large size, suggesting fusion with each other. Principal cells play a key role in the removal of proteins and other substances from the lumen via an endocytic apparatus, in conjunction with the more actively endocytic clear cells [13,59,73–75] The ongoing endocytic activity in KO mice coinciding with the abundance of lysosomes suggests that the endocytic activity of principal cells is a highly efficient system and not regulated by a negative feedback mechanism. In addition, the accumulation of these lysosomes in principal cells appeared to affect the integrity of the Golgi apparatus and ER cisternae, which as highly active secretory cells could have an impact on sperm maturation. Principal cells are the predominant cell type and actively involved in the secretion of proteins that bind to sperm and contribute to their maturational state [8,9,76]. Remodeling of the sperm surface during epididymal transit from caput (immature) to cauda (mature) has been shown to be accompanied by a substantial proteomic streamlining of proteins [77] involving a major role of its epithelial cells to endocytose these proteins from the epididymal lumen.

Lysosomal appearance and accumulation in *Naglu*-/- mice reflects the characteristic lysosomal storage phenotype observed in other lysosomal storage diseases, such as for the male reproductive tract of *Hgsnat* KO mice [49] as well as in different tissues and organs of the body [38–40,78]. The marked increase in LAMP2, cathepsin B and prosaposin immunoreactivity throughout the epididymal epithelium of *Naglu* -/- mice provides additional evidence of the extensive lysosomal accumulation observed ultrastructurally. In eMPs, the presence of intense cathepsin B immunoreactivity and prosaposin within the enlarged lysosomal compartment further supports its lysosomal nature. The observation that Cathepsin B and LAMP2-positive lysosomes did not overlap across the different epithelial cell types coincides with the well documented cell type specific expression of lysosomal enzymes in the epididymal epithelium [79–81]. Taken together the structural abnormalities of epithelial cells in *Naglu* KO mice suggest that the proposed male infertility reported from six months of age onward [50] could in part be due to lysosomal accumulation and defective proteoglycan turnover by principal cells indirectly impacting sperm reproductive capacity.

Interestingly, differences in the structural appearance of lysosomes of the caput region and compared to those of the initial segment and corpus suggested region specific uptake of substrates along the epididymal duct. Additionally, the selective appearance of uniquely structured lysosomes within the two different subtypes of principal cells of the caput suggested intraregional differences in substrate uptake by these cells. Regional specific differences for principal and other epithelial cells in terms of their structural and functional parameters have been amply reported along the length of the duct [8,9,26,82–88]. Indeed, differences in the staining properties for principal cells, referred to as the checkerboard pattern, suggested varying degrees in the ability of individual principal cells to secrete proteins within a given epididymal region [89,90]. These findings as well as our current data imply that not all principal cells are synchronized to function in precisely the same manner in any given region of the epididymal duct, an avenue for future investigations.

Notably, the structural features of the content of the lysosomes of principal cells of the caput region resembled that of the cristae of mitochondria of these cells. Indeed, abnormal structurally altered mitochondria were noted in principal cells of the caput region and images suggested a link between such mitochondria and lysosomes whereupon the two fused with one another. In addition, lysosomes of principal cells revealed small membranous discs in side views not unlike the cristae of their mitochondria, as well as small disc-like fingerprint impressions in face views, a link that requires further investigation. Curiously, such observations were restricted to the caput region only. In contrast, principal cells of *Hgsnat-/-* mice did not reveal region specific phenotypes of their lysosomes. In fact, principal cells in all regions revealed only pale lysosomes, and there were no signs of small discs or disc-like fingerprint impressions in any lysosomes of principal cells in any epididymal region [49].

### *Naglu* gene inactivation reveals ultrastructural alterations to eMPs

In the present study, cells with specific immunological markers (CD68+/Moma2+/CX3CR1+ cells) were characterized as eMPs and lodged in the basal compartment of the epididymal epithelium of *Naglu* KO mice. While CD68-positive macrophages have not been demonstrated in the epididymis of mice using LM-IHC, a high expression of CD68 mRNA was noted in epididymal macrophages of the mouse [91,92].

By TEM, eMPs of *Naglu* -/- mice from the initial segment to the distal corpus revealed a cytoplasm that contained a vast accumulation of pale lysosomes of varying sizes and shapes with some of gigantic size. Some eMPs appeared to be undergoing degeneration revealing damage of their plasma membrane resulting in liberation of their contents into the adjacent lateral intercellular space.

The accumulation of a vast number of pale lysosomes in eMPs suggested that these immune cells are active in the phagocytosis of substances with a heparan sulfate signature derivation, arising from the surrounding milieu of the epididymal epithelium. A phagocytic role for eMPs is validated by the presence of similar sporadic isolated membranous profiles and numerous small plaques of electron dense material often of spherical shapes.

Indeed, TUNEL staining was markedly increased in *Naglu-/-* compared with WT animals. TUNEL-positive labeling was observed in the epithelium and seen as a punctate cytoplasmic reaction in some principal cells and more diffuse, stronger reaction in an occasional basal cell. However, particularly strong immunostaining was observed within the large lysosomal compartments of eMPs. However, the nuclei of a few of these cells were not always reactive; albeit the nucleus was not readily apparent in most eMPs. This suggested that despite their abnormal structural appearance a few eMPs were not undergoing apoptosis. The presence of TUNEL-positive material within their enlarged lysosomal compartments may correlate with the presence of patches of irregularly shaped electron dense bodies noted within the interior of these structures. These distinct patches may correspond to fragmented DNA of epithelial cells phagocytosed by eMPS, but future studies are needed to substantiate this hypothesis.

The discovery of a functional population of eMPs in the epididymal epithelium has been demonstrated where they perform key roles in the preservation of epithelial integrity by elimination of extraneous harmful debris and limiting excessive immune activation upon infection [26,27,29,93–95]. Taken together, such observations suggest that in our KO mouse model, eMPs are actively phagocytosing degenerating principal cells and their contents, but in the absence of NAGLU, become congested with lysosomes unable to degrade HSPGs. The inability of eMPs to perform their role in protecting and defending the epithelial cells from harmful substances could be considered as another factor rendering an infertile status to *Naglu-/-* mice.

A link of the M/M population of eMPs of the epididymis can be made with the resident mononuclear microglia of the central nervous system which are renowned for immune surveillance and phagocytic activity. In MPS IIIB mouse models, microglia exhibited an overexpression of CD68 and MOMA-2, along with marked expansion of the lysosomal compartment linked to an “activated” microglial state with pro-inflammatory properties [96]. This phenotype in the brain reflects an overload of undegraded substrates typical of lysosomal disorders in other systems and as noted for *Hgsnat -/-* mice [49,97] and for absence of NAGLU in the present study.

### Identification of eMPs of *Naglu-/-* mice

In the literature, M/M cells have been shown to be comprised of different subsets based on specific markers [26–29,98,99]. Notably, early TEM analysis demonstrated that a subset of halo cells comprised a population of “circulating” wandering M/M cells residing in the epithelium without attachment to the basement membrane [8,13]. However, M/M cells have been reported to contact the basement membrane by LM confocal imaging [24,100]. Our recent TEM analysis has revealed a population of M/M cells firmly anchored to the basement membrane constituting a “resident” population of M/M cells [101], as demonstrated in many other tissues and organs of the body [60, 102, 103]. These observations suggest that M/M cells function in a protective role for the epithelial cells and sperm both at the level of recruitment from the circulation as well as being a nonmobile stable component of the epithelium.

Additionally, the present study also revealed that some eMPs had a close affiliation with small spherical halo cells having a small cytoplasmic to nuclear ratio and morphology characteristic of T-lymphocytes described in the literature [8,20]. Studies on dendritic cells in other tissues have demonstrated a close relationship with T-lymphocytes [104]. In our recent TEM analysis of the epididymal epithelium, we noted that a close association of T cells with eMPs [101]. In the epididymis, Da Silva et al., 2011 [24], demonstrated that isolated epididymal dendritic cells presented ovalbumin to T cells *in vitro* regulating an interplay between immune tolerance and activation, fundamental to male fertility. Hence the proximity of T cells with eMPs suggests that in the *Naglu* KO mouse, T cells play a key role in the activation and infiltration of dendritic cells, thereby enhancing the clearance of accumulated degraded material from the intercellular space.

### Structural features of zebra bodies of eMPs

One of the intriguing aspects of the *Naglu -/-* mouse model was the finding of zebra bodies in the pale-stained lysosomes of eMPs. Structurally, zebra bodies resembled the cristae of mitochondria of adjacent principal cells. Given that principal cells appear to undergo degeneration and that eMPs function as phagocytic cells, it is reasonable to assume that the organelles of the degenerating cells end up in their lysosomal system. While eMPs exhibited zebra bodies, this was in direct contrast to the M/M cells occupying the interstitial space of the testis and those of the intertubular spaces of the epididymis. Such a finding suggests their presence has to do with an internalized substrate related with the epididymal epithelium.

Interestingly, zebra bodies have been demonstrated in neuronal lysosomes of the cortex of brains of MPS IIIB mouse models where several unrelated metabolites, including subunit c of mitochondrial ATP synthase (SCMAS) of mitochondria localized [50,105]. In the present study, SCMAS immunoreactivity was detected within enlarged lysosomal compartments of eMPs of KO mice and seen as several large reactive bodies which may be related to the zebra bodies observed ultrastructurally in eMPs. Taken together these findings provide additional evidence for the uptake of mitochondria of principal cells within the lysosomal compartments of eMPs, as noted in lysosomes of neurons of MPS IIIB mouse. In comparison, prior studies in murine MPS IIIC (*Hgsnat-/-*mouse) models reported major lysosomal accumulations in epididymal eMPs, but with total absence of zebra bodies [49], indicating that although both mucopolysaccharidoses share lysosomal dysfunction, the morphology and composition of stored material vary depending on the defective enzyme.

### Different basal cell phenotypes in *Naglu-/-* mice

By TEM analysis, basal cells of *Naglu-/-* mice presented different phenotypes. One subtype presented a cytoplasm filled with pale lysosomes and were defined as distended flattened, elongated cells spanning some distance along the basement membrane; they resembled those described by Veri et al. [106]. Other basal cells with a distended appearance had a preferential orientation directed towards the apical luminal aspect of the epithelium [57,106,107]. In either scenario, basal cells displayed prominent dilated pale-stained lysosomes suggestive of a lysosomal disorder and their capacity to function in endocytosis [8,13,15]. Interesting, while their pale lysosomes displayed membranous profiles and electron dense aggregates, they never contained zebra bodies.

Conversely, some basal cells did not exhibit an abnormal altered appearance nor a cytoplasm containing pale lysosomes. They had features of primitive stem cells as described in our recent study [101]Over the past decade, various studies have demonstrated that a population of basal cells possess stem cell capability [52,58,91,108,109], as indicated by their ability to form epididymal organoids [110].

Recent studies using single cell RNAseq in adult mice have proposed the existence of several subtypes of basal cells [91,111], a finding correlating with our TEM morphological description of different subtypes of basal cells in normal rodents [101]. Taken together the different data support the existence of subsets of basal cells, which in *Naglu-/-* mice display distinct phenotypic features, including the apparent resistance of a primitive stem cell-like population to the effects of NAGLU deficiency.

In conclusion, our findings on NAGLU deficiency highlight how lysosomal dysfunction drives structural and functional impairment in both epididymal epithelial cells and eMPs, compromising sperm maturation and local immune regulation. Moreover, the data provide important insight into the role of eMPs and epithelial cells in maintenance of the epididymal environment for sperm maturation and underscoring the essential role of heparan sulfate metabolism in male fertility.

## Supporting information

S1 Fig Validation of NAGLU deficiency in reproductive tissues. (A, B) NAGLU enzymatic activity measured in testis (A) and epididymis (B) from WT and *Naglu-/-* mice (n = 4 per genotype for each tissue). Data are presented as mean ± SEM. Statistical analysis was performed using a two-tailed unpaired Student’s t-test. (C, D) Immunocytochemical detection of heparan sulfate (magenta) with DAPI counterstaining (blue) in the epididymis of WT (C) and *Naglu-/-* (D) mice. Heparan sulfate accumulation is detected throughout the epididymal epithelium of *Naglu-/-* mice, including different epithelial cell types. Lu, lumen; IT, intertubular space; P, principal cells; eMP, epididymal mononuclear phagocyte. Scale bars are indicated in each panel.
(TIF)

S2 Fig TEM analysis of WT (A) and *Naglu*-/- mice (B-D) of seminiferous tubules and interstitial space (IS) of the testis. In KO mice (B-D), the seminiferous epithelium consists of a normal complement of different generations of germ cells (GC) and Sertoli cells (S) as seen in WT mice (A). An intact blood testis barrier (BTB) between adjacent Sertoli cells is noted and a well-developed acrosome (Ac) overlies the nucleus of spermatids. While few small to moderate size homogeneous dense lysosomes (arrowheads) are evident in the cytoplasm of Sertoli cells of WT and KO mice (compare A with B-D), in KO mice (B-D) only the occasional larger dilated pale stained lysosome (Ly) is noted containing loose membranous profiles (B, arrows) and whorls or plaques of electron dense material of varying shapes and sizes (C, D circles); prosaposin antibody confirms lysosomal staining by LM-IHC (arrows, C inset). Myoid cells (My) of the lamina propria contain an abundance of pale lysosomes (D inset), as do macrophages (M) of the interstitial space (IS) (B, D inset). N, nucleus of Sertoli cells. Scale bars: 2 μm.
(TIF)

S3 Fig LAMP2 and cathepsin B immunofluorescence in the epididymis of WT and *Naglu*-/-mice. (A) WT epididymis and (B) *Naglu*-/- epididymis showing LAMP2 (green), cathepsin B (CatB; red), and DAPI nuclear staining (blue). In *Naglu*-/- mice, LAMP2 and CatB immunoreactivity are markedly increased throughout the epididymal epithelium compared with WT mice. The inset shows an eMP at higher magnification, illustrating intense CatB immunoreactivity within the enlarged lysosomal compartment and the sparse reaction of LAMP2 immunoreactivity within the eMP. In *Naglu*-/- mice, principal cells (P) contain numerous LAMP2-positive compartments (Ly) of varying sizes, with increased CatB immunoreactivity that does not always coincide with LAMP2-positive structures. Lu, lumen; IT, intertubular space; P, principal cells; eMP, epididymal mononuclear phagocyte; CC, clear cells; BC, basal cells; My, myoid cells; M, intertubular macrophages; arrows indicate eMPs. Scale bars are indicated in each panel.
(TIF)

S4 Fig EM images of principal cells (P) of the caput region of the epididymis of KO mice (A, B) highlighting at higher power the different lysosomal (Ly) phenotypes (blue boxes: A1-4, B1 and purple boxes: A-1–4) and mitochondria of P cells (boxed in red: A1-4, B1-3). Lysosomes of P cells with a moderate to densely stained matrix display small clusters of flattened membranous discs (stars) aligned parallel to each other, as seen in side views, and appearing as fingerprint impressions in face views, along with small vesicles and a deeply stained appearance (blue boxes: A-1–4, B-1). Lysosomes of P cells with a pale-stained matrix show few aggregates of membranous discs (stars, purple boxes: A-1–4) and occasional membranous profiles (arrows) and plaques of electron dense material of varying shapes and sizes (circles). The lysosomes of both P cell populations reveal intimate contact and fusion with one another (F-Ly) (purple box: A-1–4). Images of mitochondrial atrophy (m) are noted in both populations of P cells of the caput region (red boxes A-1–4), as described in the text. Lysosomes associated with such mitochondria display fingerlike impressions (stars, B-1, B-2). N, nucleus; CC, clear cell; My, myoid cell; E, endosome; BM, basement membrane. Scale bars: (A, B) 2 μm; (blue, purple and red A-1/B-3) 200 nm.
(TIF)

S5 Fig TEM features of eMPs of KO mice of the initial segment (A), and caput (B-D) regions of the epididymis. In (A), a lower power view of eMPs, shown in Figure 8A, reveals the expansive nature of these cells containing giant (GL) and gigantic lysosomes (Gly) and distinct separations from the basement membrane (BM) by P cells or contact with it (curved arrow). An elongated apically oriented basal cell (BC) filled with small pale lysosomes contacts the basement membrane (BM). Dilated lateral intercellular spaces (LIS) between adjacent P cells demonstrate vesicular and membranous profiles. In (B, C), eMPs are anchored to the BM and reveal giant (GL) lysosomes. When present, the nucleus (N) of eMPs is highly indented by large lysosomes. In (B), P cells show an abnormal mitochondrion (boxed in) and some pale lysosomes contain small disc-like fingerprint impressions (stars). In (D), two BCs display pale lysosomes and irregularly shaped nuclei (N). Overlying BCs is an eMP housing moderately stained lysosomes. While BCs firmly adhered to the BM, the eMP is separated from it by thin processes (arrowheads) of these cells. P cells reveal irregularly shaped discoid nuclei (N) and moderately stained lysosomes containing small disc-like fingerprint impressions (stars). Zebra bodies (asterisks) are noted in eMPs (A-D), but not in basal cells. Scale bars = 2 μm.
(TIF)

S6 Fig SCMAS labeling in the epididymis of *Naglu-/-* mice. (A) SCMAS (red) immunostaining with DAPI counterstaining (blue). SCMAS is detected throughout the epididymal epithelium. Within eMPs (arrows), SCMAS-positive structures appear larger than those observed in other epithelial cells. The inset of A shows a higher-magnification view of an eMP. In this merged panel, the blue channel is displayed at a saturated exposure solely to facilitate visualization of the eMP boundary, which is delineated by a white line based on its characteristic morphology and position within the epididymal tissue of *Naglu-/-* mice. Lu, lumen; IT, intertubular space; N, nucleus; My, myoid cells; P, principal cells; eMP, epididymal mononuclear phagocyte. Scale bars are indicated in each panel.
(TIF)

S7 Fig EM examination of the intertubular space (IT) of the epididymis of WT (A) and KO (B, C). In (B) a large macrophage contains a massive accumulation of pale lysosomes (Ly) that fill its cytoplasm, compared to WT mice (A) showing few small dense lysosomes. The pale lysosomes of KO mice are moderate in size and devoid of content; some show signs of fusion (F-Ly). In (C), two macrophages (M) of the IT space reveal numerous pale lysosomes (Ly) along with one that does not. Two elongated BCs containing lysosomes (Ly) adhere to the basement membrane (BM). P cells display pale lysosomes. My, myoid cells, IT, intertubular space. Scale bars = 2 μm.
(TIF)

S8 Fig (A–C) TUNEL assay (green) with DAPI counterstaining (blue). P cells show small punctate TUNEL-positive structures (arrowheads). The three panels to the right of C show higher-magnification inset of an eMP. In the merge TUNEL+/DAPI panel, DAPI signal is displayed using a saturated blue exposure to facilitate visualization of the cell boundaries. The white line delineates the boundary of the eMP shown in the inset, based on its characteristic morphology and position within the epididymal tissue of *Naglu-/-* mice. The TUNEL channel (green) shows TUNEL-positive material within the lysosomal compartment of the eMP (white circles), while its nucleus (N) is not TUNEL-positive. The DAPI-only panel, shown without modification of the blue-channel exposure, demonstrates diffuse DAPI-positive material colocalizing with the TUNEL-positive structures (white circles) suggested to be DNA fragments of phagocytosed P cells. Lu, lumen; IT, intertubular space; BC, basal cells; arrows indicate eMPs. Scale bars are indicated in each panel.
(TIF)
